# Diabetes and its complications: molecular mechanisms, prevention and treatment

**DOI:** 10.1038/s41392-025-02401-w

**Published:** 2026-01-19

**Authors:** Lijun Zhao, Jiamin Yuan, Qing Yang, Jing Ma, Fenghao Yang, Yutong Zou, Ke Liu, Fang Liu

**Affiliations:** 1 Department of Nephrology, West China Hospital of Sichuan University, Chengdu, China; 2 Laboratory of Diabetic Kidney Disease, Kidney Research Institute, Department of Nephrology, West China Hospital, Sichuan University, Chengdu, China; 3 Department of Clinical Medicine, Southwest Medical University, Luzhou, China

**Keywords:** Metabolic disorders, Endocrine system and metabolic diseases

## Abstract

Diabetic complications represent a formidable clinical challenge characterized by hyperglycemia-induced multiorgan dysfunction and dysregulated intercellular signaling networks. Advances in spatial multiomics and single-cell transcriptomic techniques, along with insights into aberrant signaling via myokines, cytokines, hormones, the gut microbiota, and exosomes, have revealed the molecular heterogeneity and dynamic inter-organ crosstalk underlying diabetes. Digital diabetes prevention programs have demonstrated effectiveness in high-risk populations through the use of remote tools to support lifestyle changes, reduce hemoglobin A1c, and delay the onset of type 2 diabetes. The therapeutic landscape for diabetic complications has been reshaped by agents with proven cardiorenal benefits, including sodium‒glucose cotransporter 2 inhibitors, glucagon‒like peptide-1 receptor agonists, and nonsteroidal mineralocorticoid receptor antagonists, with combination therapies offering potential additive or synergistic effects. However, their optimal application requires careful benefit–risk assessment across diverse patient populations. Novel therapeutic strategies involving mesenchymal stem cells and their derived exosomes, gut microbiota modulation, bioactive compounds from traditional Chinese medicine, and AI-assisted disease management systems offer promising approaches to correct molecular dysfunctions. This review summarizes recent advances in the mechanisms, prevention, and treatment of diabetic complications, alongside a critical examination of current bottlenecks in translational applications. The remaining challenges include establishing long-term safe regenerative therapies and effectively integrating AI into clinical workflows. Although AI shows promise, issues such as limited data diversity and low model interpretability hinder its generalizability and clinical trust. Addressing these challenges will be essential for transitioning toward a proactive, personalized, and patient-centered model of care.

## Introduction

Diabetes mellitus (DM) refers to a group of metabolic disorders primarily characterized by hyperglycemia due to absolute or relative insulin deficiency, impaired insulin action, or both.^1^ It was estimated that in 2022, there were 828 million adults worldwide with diabetes, representing a marked increase of 630 million compared with 1990, with a prevalence rate of 13.9% for women and 14.3% for men.^2^ The number of cases is projected to exceed 1.31 billion by 2050^3^, thus imposing a significant burden on both healthcare and the global economy.^4^ However, the increasing incidence of diabetes has not been accompanied by a corresponding rise in its treatment; this is particularly apparent in low- and middle-income nations, where research reveals that 59% of diabetic patients worldwide aged 30 years and above are not receiving treatment.^2^

**Fig. 1:**
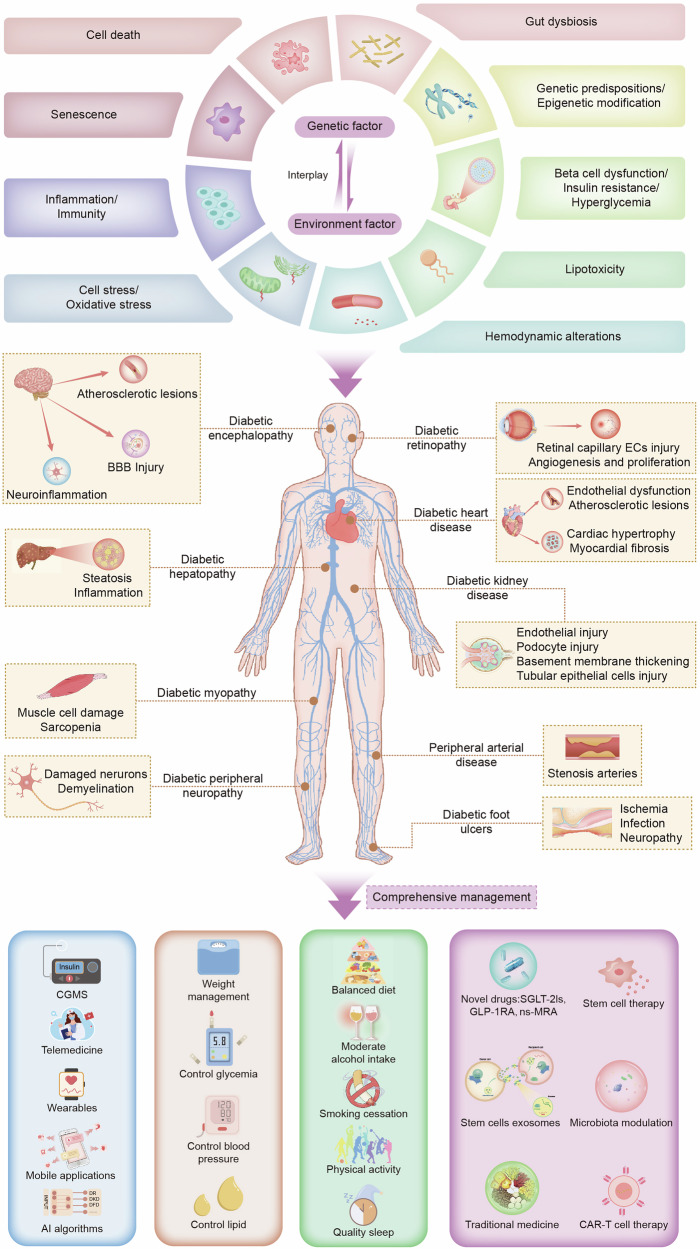
Schematic representation of the mechanisms and co-management strategies of diabetic complications. The interplay between genetic and environmental factors gives rise to the development of diabetes and its complications. The mechanisms involved include beta cell dysfunction, insulin resistance, hyperglycemia, genetic predisposition, epigenetic modifications, changes in the gut flora, cell death, cellular senescence, inflammation, immunity, cell stress, oxidative stress, hemodynamic alterations, and lipotoxicity. The complications of diabetes affect multiple organ systems, including the renal, cardiovascular, cerebral, peripheral vascular, ophthalmic, hepatic, muscular, and nervous systems, as well as the feet. The prevention of diabetic complications necessitates the active collaboration of individuals, families, communities, and healthcare institutions, alongside the implementation of comprehensive co-management and co-treatment strategies to effectively address these multifaceted issues. BBB blood–brain barrier, CAR-T Chimeric antigen receptor T, CGMS continuous glucose monitoring system, ECs endothelial cells, ns-MRAs nonsteroidal mineralocorticoid receptor antagonists, GLP-1RAs glucagon-like peptide-1 receptor agonists, SGLT-2Is sodium‒glucose cotransporter 2 inhibitors

Chronic hyperglycemia induces systemic metabolic disturbances that drive both macrovascular atherosclerosis and microvascular injury across cardiac, cerebral, renal, and peripheral circulation. This constellation of pathology is collectively termed “diabetic panvascular disease (DPD),” reflecting common molecular mechanisms and interdependent risks among vascular complications.^5^ Recent studies have emphasized the dynamic interplay of systemic and tissue-specific risk and protective factors in the development of diabetic complications.^6^ This review comprehensively examines the molecular mechanisms underlying diabetic complications across multiple organs, as well as current prevention strategies and recent multi-organ therapeutic approaches (Fig. 1). These frameworks provide a foundation for improving the management of diabetic complications and emphasize the importance of adopting comprehensive treatment approaches to address the multifaceted challenges associated with diabetes.

## Molecular mechanisms and mediators of organ crosstalk in diabetic complications

Diabetes includes several forms, namely, type 1 diabetes (T1D), an early-onset autoimmune condition; type 2 diabetes (T2D), a late-onset non-autoimmune form accounting for more than 90% of cases^7^; and monogenic diabetes, such as Maturity-Onset Diabetes of the Young, a rare inherited form resulting from a single-gene defect; neonatal diabetes; gestational diabetes associated with pregnancy; and latent autoimmune diabetes in adults,^8^ an autoimmune condition occurring in adulthood. As reviewed elsewhere,^9^ the onset of diabetes results from a complex interplay of genetic and environmental factors, with T2D accounting for 96% of cases.^10^ In this review, we focus on recent important advances in the understanding of the pathology of T2D and its complications.

β cells are recognized as central nodes in pathways that mediate hyperglycemia. Diabetes is characterized by a combination of β-cell dysfunction and insulin resistance in the liver and muscles, representing key features of T2D. Gene editing, particularly CRISPR-Cas9, holds the potential for the precise differentiation of stem cells into β cells.^11–13^ Furthermore, manipulating the lncRNA MIR503HG or ZnT8 in stem cell-derived pancreatic progenitors improved insulin synthesis and secretion.^14,15^ As the understanding of β-cell biology advances, a combination of stem cell therapy and gene editing presents promising prospects for diabetes treatment, although further evaluation is needed to determine long-term efficacy and safety.

In addition, factors such as lipotoxicity, defects in the incretin system, hyperglucagonemia, increased renal glucose reabsorption, and central insulin resistance contribute to the progression of diabetes. These factors are collectively known as the "ominous octet".^16^ The development of diabetic complications arises from a complex interplay of metabolic dysregulation and injury mechanisms. The shared core pathways include hyperglycemia, dyslipidemia, hemodynamic alterations, oxidative stress, the formation of advanced glycation end products (AGEs) and chronic inflammation.^5,17^ Endothelial cells (ECs), key mediators of vascular lesions, absorb excess glucose *via* insulin-independent pathways, such as glucose transporter 1-3 (GLUT1-3), resulting in elevated intracellular glucose levels.^18^ Cellular hyperglycemia disrupts mitochondrial oxidative phosphorylation, fatty acid metabolism, and key signaling pathways crucial for metabolic stress adaptation, tissue integrity, and immune responses in diabetic complications.^19–21^ Metabolic reprogramming, characterized by a shift from mitochondrial oxidative phosphorylation to glycolysis, increases the production of toxic byproducts and reactive oxygen species (ROS).^22^ Moreover, the interplay between ER stress and mitochondrial dysfunction at mitochondria-associated ER membranes exacerbates intracellular calcium imbalance, alterations in mitochondrial dynamics, ROS overproduction, and apoptosis.^23–25^ In hyperglycemia, innate immune system activation, particularly through nucleotide-binding oligomerization domain-like receptor protein 3 (NLRP3) inflammasomes,^26^ promotes the release of pro-inflammatory factors, aggravating chronic inflammation, immune senescence,^27^ vascular damage, and target organ injury.^28–30^ Endothelin-1 (ET-1) is a strong vasoconstrictor that promotes inflammation, hypertrophy, and fibrosis in the heart, vessels, and kidneys.^31^ It primarily signals through ET_A_ receptors (ET_A_Rs) on vascular smooth muscle, triggering inflammation and cell growth.^32^ ET-1 can also induce vasodilation via ET_B_ receptors (ET_B_Rs) by stimulating nitric oxide and prostacyclin release from ECs. These mechanisms collectively drive inflammatory responses, cellular damage, tissue fibrosis, and progressive organ dysfunction in diabetic complications.

### Diabetic kidney disease

**Fig. 2:**
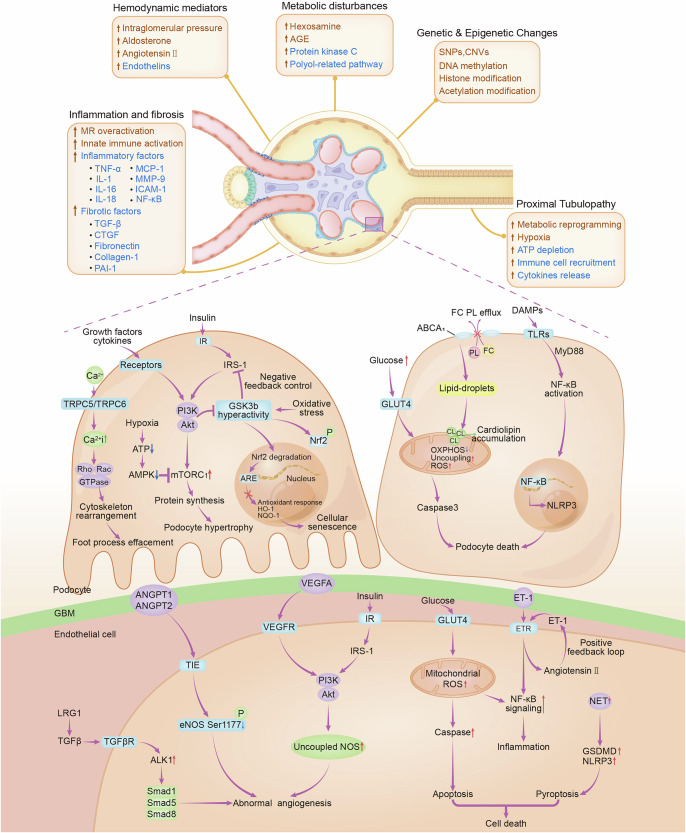
Mechanisms Underlying the Development of Diabetic Kidney Disease. Schematic overview of the drivers of glomerulopathy and tubulopathy in DKD. Early TRPC5/6-mediated Ca²⁺ influx leads to the effacement of podocyte foot processes. Activation of the PI3K/Akt/mTORC1 pathway promotes podocyte hypertrophy, with hypoxia further enhancing mTORC1 activity. Diabetes-induced oxidative stress drives GSK3β hyperactivity, reducing nuclear Nrf2 accumulation and impairing the expression of antioxidants (HO-1 and NQO1), thereby promoting podocyte senescence. Desensitized insulin signaling impairs GLUT4 translocation and glucose uptake, leading to reprogramming of glucose metabolism and mitochondrial dysfunction, which are characterized by decreased OXPHOS, increased uncoupling, and elevated ROS production. ABCA1 deficiency exacerbates cholesterol accumulation and mitochondrial damage in podocytes. Inflammation is amplified by DAMP-induced activation of the NLRP3 inflammasome and NF-κB. Endothelial dysfunction—via LRG1/TGFβ signaling, ANGPT/VEGFA imbalance, and NET deposition—further promotes abnormal angiogenesis, cytoskeletal disruption, and GBM thickening. Abbreviations: ABCA1 ATP-binding cassette subfamily A member 1, ANGPT-1 angiopoietin-1, ANGPT-2 angiopoietin-2, Akt protein kinase B, ALK1 activin receptor-like kinase 1, Ang II angiotensin II, ARE antioxidant responsive element, DKD diabetic kidney disease, ATP adenosine triphosphate, AMPK AMP-activated protein kinase, DAMPs damage-associated molecular patterns, eNOS endothelial nitric oxide synthase, ET-1 endothelin, ETR endothelin receptor, FC free cholesterol, GLUT4 insulin sensitive glucose transporter 4, GBM glomerular basement membrane, GSK3β glycogen synthase kinase 3β, GSDMD gasdermin D, HO-1 heme oxygenase-1, IR insulin receptor, IRS-1 insulin receptor substrate-1, LRG1 leucine-rich alpha-2 glycoprotein 1, MyD88 myeloid differentiation factor 88, mTORC1 mechanistic target of rapamycin complex 1, NET neutrophil extracellular traps, NLRP3 nucleotide-binding domain (NBD), LRR leucine-rich repeat, and PYD pyrin domain-containing protein 3, NF-κB nuclear factor κB, NQO1 NAD(P)H quinone dehydrogenase-1, Nrf2 nuclear factor erythroid 2-related factor 2, OXPHOS oxidative phosphorylation, P phosphorylation, PI3K phosphatidylinositol 3-kinase, PL phospholipid, ROS reactive oxygen species, Rho Ras homology, TLRs toll-like receptors, TGFβ transforming growth factor beta, TRPC5/6 transient receptor potential channel 5/6

Diabetic vasculopathy can be broadly classified into macroangiopathy and microangiopathy.^5^ As hyperglycemia progresses, patients are prone to develop pathological changes such as endothelial dysfunction and thickening of the vascular basement membrane—key features of diabetic microangiopathy. Approximately 22–40% of diabetes patients develop DKD, making it the leading cause of end-stage kidney disease, which requires dialysis or transplantation and poses a significant public health challenge.^33,34^ DKD is driven by a cascade of hemodynamic disturbances, dysregulated metabolism, and inflammatory and fibrotic processes, along with epigenetic changes (Fig. 2). Early features of DKD include intraglomerular and single-nephron hyperfiltration,^35^ driven by systemic hyperglycemia and increased angiotensin II release through tubuloglomerular feedback.^10,36^ Single-cell RNA sequencing of kidney biopsies from T2D DKD patients revealed a 1240-gene signature associated with hyperfiltration, highlighting endothelial stress and interactions between endothelial and mesangial cells.^37^ These hemodynamic changes impose additional intraglomerular wall tension and shear stress on podocytes, increasing the oxygen demand in tubular ECs to support reabsorption.^36^ Intracellular calcium levels, regulated by transient receptor potential channels, modulate Rho and Rac proteins and activate pathways associated with mechanical stretching, including the YAP/TAZ pathway, which collectively drives the reorganization of the actin cytoskeleton in podocytes.^38^ Mammalian target of rapamycin complex 1 (mTORC1)-mediated podocyte hypertrophy in response to growth factor and insulin signaling increases vulnerability to further injury.^39^ These changes lead to podocyte stress, mesangial expansion, glomerular basement membrane thickening, glomerulosclerosis and tubulointerstitial fibrosis.^40^

Podocyte metabolism undergoes early shifts in DKD, with oxidative stress promoting podocyte apoptosis.^41^ Podocyte-specific deletion of Abca1 (Abca1fl/fl) is associated with cardiolipin-driven mitochondrial dysfunction, predisposing mice to DKD.^42,43^ Cholesterol-enriched lipid droplet formation in podocytes, combined with dysregulated insulin signaling and hyperglycemia, exacerbates podocyte death and detachment.^44^ Moreover, hyperglycemia-induced oxidative stress, AGEs, and chronic inflammation drive glomerular cell senescence through glycogen synthase kinase 3β (GSK3β)-modulated nuclear factor erythroid 2-related factor 2 (Nrf2) signaling, impairing repair and worsening inflammation and fibrosis.^45^ Common oral glucose-lowering agents, including metformin,^46^ dapagliflozin,^47^ and glucagon-like peptide-1 receptor agonists (GLP-1RAs),^48^ have demonstrated efficacy in mitigating DKD-associated senescence. Furthermore, DNA damage repair and epigenetic modifications in the promoter regions of *NEPH1* and *RCAN1* have been shown to restore an intact slit diaphragm in diabetic podocytes in human samples.^49^ Loss of podocytes remains a critical factor in glomerulosclerosis, a hallmark of DKD progression^41^ (Fig. 2). Crosstalk between podocytes and endothelial cells leads to endothelial dysfunction under hyperglycemic conditions. Decreases in the ratios of angiopoietin-1 (ANGPT1) and ANGPT2, abnormal podocyte expression of vascular endothelial growth factor (VEGF), and podocyte/endothelial cell-derived ET-1 induce abnormal angiogenesis by promoting the proliferation and migration of ECs, together with tube formation.^50^ Furthermore, abnormal VEGF levels impair angiogenesis and lymphangiogenesis, contributing to renal vascular dysfunction,^51^ whereas lymphatic dysfunction exacerbates interstitial edema and fibrosis.^52^

Recent research has shifted focus from a “glomerulocentric” model to a “proximal tubulopathy” perspective. Genome-wide association studies have linked elevated expression of the *AKIRIN2* and *DCLK1* genes to renal fibrosis.^53^ Tubular epithelial cells are particularly susceptible to glucose-induced metabolic derangements.^54^ Proximal tubular hypertrophy, a compensatory response to chronic hyperglycemia, triggers metabolic reprogramming, hypoxia, adenosine triphosphate (ATP) depletion, immune cell recruitment, and cytokine release.^55^ This hypertrophic response involves activation of the adenosine 5′-monophosphate-activated protein kinase (AMPK) pathway, further exacerbating hypoxia and ATP depletion.^56^ As the disease progresses, defects in fatty acid oxidation due to the repression of transcription factors, such as sterol regulatory element-binding proteins and peroxisome proliferator-activated receptor-γ, result in energy depletion and the release of mitochondrial RNA/DNA, activating inflammatory pathways involving interferon regulatory transcription factor and transforming growth factor-beta (TGF-β).^55,57^ These injured or profibrotic tubular cells recruit macrophages, lymphocytes, and fibroblasts, promoting tissue fibrosis and leading to irreversible kidney damage.^10^

Increasing evidence highlights the important role of innate immune activation, particularly the complement system, in DKD-related inflammation.^58,59^ Interactions between innate immune components such as Toll-like receptors (TLRs) and endogenous danger-associated molecular patterns (DAMPs) or pathogen-associated molecular patterns induce nuclear factor-κB (NF-κB)-mediated inflammation^59^ while also promoting renal apoptosis and fibrosis.^60^ This establishes a vicious cycle that exacerbates kidney damage and contributes to proteinuria in individuals with diabetes.^61^ Moreover, activation of the NLRP3 inflammasome by metabolic stress and oxidative damage amplifies inflammation via IL-1β and IL-18 secretion.^62^ This inflammatory environment facilitates the recruitment of immune cells, including macrophages and T cells, which sustain kidney injury and fibrosis.^58^ Complement components such as C3, C4c, C5, and C7 are upregulated in glomeruli,^63–67^ activating both the classical and alternative complement pathways and generating anaphylatoxins (C3a and C5a). C3a/C5a receptor antagonists mitigate endothelial–to–mesenchymal transition (EndMT) in DKD by inhibiting the WNT–β–catenin pathway, thus potentially alleviating glomerular fibrosis.^68^ Overactivation of complement pathways promotes inflammation, immune cell recruitment, and kidney injury^69^ while triggering downstream pathways involving ROS, NF-κB, and protein kinase C (PKC).^69,70^ Many researchers and clinicians believe that the objectives for the treatment of patients with diabetes and chronic kidney disease (CKD) have changed and that anti-inflammatory drugs will play an important role in the management of DKD in the future. It has even been predicted that by 2030, the focus of DKD treatment will be on reducing inflammation.^71^

“Metabolic memory” refers to the phenomenon in which early episodes of hyperglycemia leave lasting molecular imprints—such as epigenetic modifications and persistent activation of signaling pathways (e.g., PKC, NF-κB, and transforming growth factor (TGF)-β)—that drive the progression of diabetic complications even after glycemic control is achieved.^72^ In DKD, prior high-glucose exposure “primes” kidney cells for persistent injury so that inflammation and fibrosis continue despite later glycemic control. The concept of metabolic memory in DKD underscores the critical role of epigenetic alterations in shaping long-term renal outcomes.^73^ Mechanistic studies have associated DNA methylation, podocyte DNA double-strand breaks, and glomerular DNA methylation with a decline in the estimated glomerular filtration rate (eGFR).^74^ Recent findings suggest that the demethyltransferases fat mass and obesity-associated protein in macrophages facilitate the transition from the proinflammatory M1 phenotype to the anti-inflammatory M2 phenotype, modulating inflammation and glycolysis through N6-methyladenosine modification of the neuronal PAS domain protein 2.^75^ These insights position epigenetic mechanisms as potential therapeutic targets for mitigating hyperglycemia-induced kidney damage.

DKD involves complex pathological processes, including glomerular hyperfiltration, podocyte dysfunction, tubular injury, and immune system activation. Emerging therapeutic approaches targeting epigenetic modulation, immunoregulation, and both glomerular and tubular pathways hold promise. Special attention to the “metabolic memory” phenomenon may further guide the development of novel interventions to reverse or prevent hyperglycemia-induced renal damage.

### Diabetes-related cardiovascular disease (CVD)

CVD remains the leading cause of mortality in individuals with T1D and T2D, accounting for 44% and 52% of deaths, respectively.^76^ Diabetes-associated CVDs, including coronary artery disease and diabetic cardiomyopathy, are commonly classified as macrovascular and microvascular complications, respectively, on the basis of the underlying pathological changes observed in DPD.

**Fig. 3:**
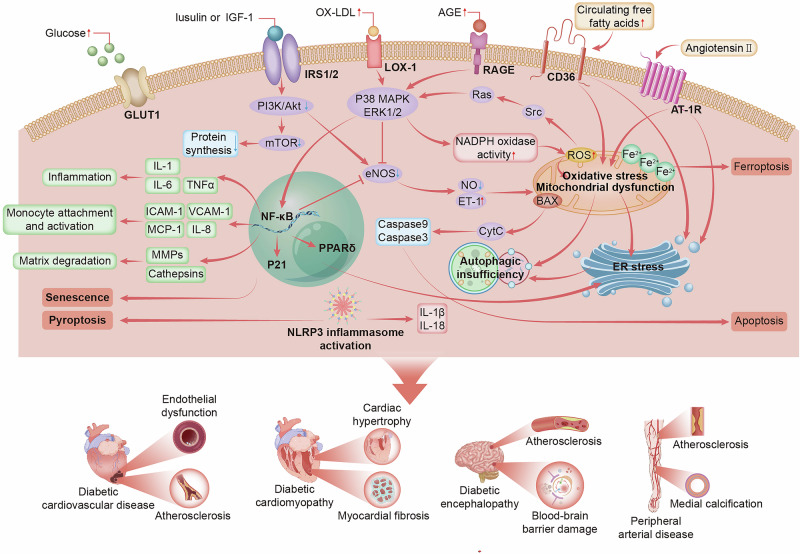
Pathology and molecular mechanisms associated with diabetes-related CVD. Endothelial cell dysfunction represents a key trigger of diabetes-related CVD. Initially, glucose enters the cell via the GLUT1 transporter. Under insulin resistance, the binding of insulin to IRS1/2 receptors is reduced, leading to decreased activity in the PI3K/AKT and mTOR signaling pathways and a decrease in protein synthesis. Concurrently, ox-LDL binds to LOX-1, activating the P38 MAPK and ERK1/2 signaling pathways, which enhance inflammatory responses. The binding of AGEs to RAGE triggers NADPH oxidase, increasing ROS production and leading to oxidative stress. Additionally, CD36 binds to circulating free fatty acids, and AT-1R binds to angiotensin II, further activating oxidative stress, mitochondrial dysfunction, and ER stress. In the cytoplasm, NF-κB promotes the expression of inflammatory cytokines and matrix degradation. Activation of the NLRP3 inflammasome facilitates the release of IL-1β and IL-18, intensifying the inflammatory response. Oxidative stress and mitochondrial dysfunction induce apoptosis and ferroptosis. ER stress exacerbates autophagy insufficiency, leading to the accumulation of intracellular waste and ultimately promoting apoptosis. These molecular mechanisms interact to cause the development and progression of complications such as diabetic cardiovascular disease, diabetic cardiomyopathy, diabetic encephalopathy, and peripheral arterial disease. Abbreviations: AGE advanced glycation end product, AMPK AMP-activated protein kinase, ATG1 Autophagy-related gene 1, AT-1R Angiotensin II type 1 receptor, BAX Bcl-2-associated X protein, CD36 cluster of differentiation 36, CytC Cytochrome C, eNOS endothelial nitric oxide synthase, ER endoplasmic reticulum, ERK1/2 extracellular signal-regulated kinase 1/2, ET-1 endothelin-1, GLUT Glucose Transporter, ICAM-1 Intercellular Cell Adhesion Molecule-1, IGF1 insulin-like growth factor 1, IL-1 Interleukin-1, IL-1β interleukin-1β, IL-18 interleukin-18, IL-6 Interleukin-6, IL-8 Interleukin-8, IRS1/2 insulin receptor substrate 1/2, MCP-1 Monocyte Chemoattractant Protein-1, MMPs Matrix Metalloproteinases, mTOR mechanistic target of rapamycin, NADPH nicotinamide-adenine dinucleotide phosphate, NF-κB nuclear factor kappa-B, NLRP3 NLR family pyrin domain containing 3, NO Nitric oxide, P38 MAPK p38 mitogen-activated protein kinase, PI3K phosphatidylinositol 3-kinase, PPARδ peroxisome proliferator-activated receptor δ, RAGE receptor for advanced glycation end product, ROS reactive oxygen species, TNFα Tumor Necrosis Factor α, VCAM-1 Vascular Cell Adhesion Molecule-1

Coronary artery disease is characterized by segmental atherosclerotic lesions affecting multiple vascular branches, reflecting widespread macrovascular involvement.^77^ In contrast, endothelial dysfunction—an early and independent predictor of cardiovascular events—contributes to both macrovascular and microvascular pathology, playing a central role in the progression of diabetic CVD^78,79^ (Fig. 3). Hyperglycemia exacerbates oxidative stress and inflammation, reducing nitric oxide (NO) bioavailability and impairing endothelial function.^80,81^ Recent findings suggest that hyperglycemia-induced “metabolic memory” in ECs represents a novel feature of endothelial dysfunction. Chronic hyperglycemia triggers NF-κB signaling, the upregulation of miR-27a-3p, the downregulation of Nrf2, the TGF-β signaling, the downregulation of miR-29, and the induction of EndMT. These changes persist even under normoglycemic conditions, contributing to perivascular fibrosis and cardiac dysfunction.^82^ EndMT plays a critical role in the development of diabetic atherosclerosis and is driven by various atherogenic stimuli, including hyperglycemia, AGEs, and oxidized low-density lipoprotein (ox-LDL). These factors induce EndMT through the activation of proinflammatory pathways and increasing oxidative stress, leading to endothelial dysfunction and plaque instability.^83^ Hyperglycemia upregulates the expression of mesenchymal markers, such as α-SMA and fibronectin, while downregulating the expression of endothelial markers, such as CD31.^84^ The AGE–RAGE axis activates the NF-κB pathway, leading to increased production of pro-inflammatory cytokines and chemokines, which further exacerbates endothelial dysfunction and facilitates the transition to a mesenchymal phenotype.^85^ Hyperglycemia also disrupts the CAV1–CAVIN1–LC3B axis, impairing autophagy and facilitating low-density lipoprotein (LDL) transcytosis, thereby accelerating atherosclerotic pathology.^86^ Ox-LDL can induce EndMT by activating the TGF-β signaling pathway and increasing ROS generation in ECs. This ultimately leads to the loss of endothelial cell integrity and the acquisition of mesenchymal characteristics, both of which contribute to plaque formation and instability.^85^ Multiomics analysis of human atherosclerotic plaques also identified several novel EndMT candidates, including USF1, PTGS2, TPM1, and FN1.^87^ Single-cell RNA sequencing (scRNA-seq) was used to identify transcriptional heterogeneity in dysfunctional ECs, revealing that EC-specific overexpression of SRY-related high mobility group box 4 promotes atherogenesis and EndMT.^88^ As key organelles in energy metabolism, mitochondria are also the primary sources of ROS that damage mitochondrial DNA.^89^ Hyperglycemia-induced mitochondrial ROS increase SIRT1-mediated PINK1/Parkin-dependent mitophagy,^90^ making mitochondrial dysfunction a potential therapeutic target to mitigate diabetes-associated atherosclerosis.

Diabetic cardiomyopathy (DCM) is characterized by ventricular dysfunction in the absence of coronary artery disease or hypertension.^91^ AGEs, formed through the reactions of proteins and lipids with high glucose levels, crosslink extracellular matrix (ECM) proteins, inhibit ECM degradation by matrix metalloproteinases (MMPs) and increase cardiac stiffness, resulting in diastolic dysfunction.^92,93^ Altered cardiac mechanics further stimulate profibrotic responses in fibroblasts and myofibroblasts through mediators such as TGF-β, tumor necrosis factor (TNF), angiotensin II, and interleukins.^94^ scRNA transcriptomics was employed to elucidate the cellular profiles of diabetic hearts, identifying nine fibroblast subsets, among which cluster 4 fibroblasts were significantly elevated in the diabetic myocardium.^95^ Moreover, hyperglycemia leads to electron leakage from the mitochondrial electron transport chain, forming superoxide ions and generating excessive mitochondrial ROS (mtROS). This accumulation of mtROS contributes to mitochondrial dysfunction, activates the NLRP3 inflammasome, and induces pyroptosis, ultimately exacerbating DCM.^95,96^ DCM appears to progress through an initial subclinical phase characterized by subtle structural and functional abnormalities (e.g., impaired diastolic relaxation), followed by severe diastolic heart failure with preserved ejection fraction (HFpEF), and ultimately progresses to systolic dysfunction presenting as heart failure with reduced ejection fraction.^97–99^ HFpEF constitutes approximately half of all heart failure cases, particularly in T2D, and is defined by a left ventricular ejection fraction ≥ 50% with predominant exercise intolerance.^100,101^ In T2D, impaired cardiomyocyte Ca²⁺ handling contributes to HFpEF pathogenesis.^102^ Concurrent ROS overproduction and AGE deposition drive concentric left ventricular remodeling and myocardial stiffness,^103^ whereas metabolic derangements (hyperglycemia, elevated free fatty acid (FFAs) and proinflammatory cytokines) exacerbate insulin resistance and impair angiogenesis.^104^ These subclinical perturbations collectively precipitate HFpEF.

Diabetes can also lead to “diabetic encephalopathy (DE),” a condition encompassing ischemic stroke, transient ischemic attacks, vascular dementia, and neurodegenerative changes. DE primarily manifests as cognitive and behavioral impairments, along with memory dysfunction.^105^ Hyperglycemia initiates a positive feedback loop involving the tyrosine kinase ErbB4 and the mammalian target of rapamycin (mTOR), contributing to tau hyperphosphorylation under hyperglycemic conditions.^106^ The PI3K/Akt/mTOR signaling pathway may also exacerbate DE by suppressing autophagy in a T2D rat model.^107^ Hyperglycemia also disrupts the structure and function of the blood–brain barrier (BBB) by inducing oxidative stress and secondary inflammatory responses, impairing brain function and the biosynthesis of neurotransmitters.^108,109^ Mitochondrial dysfunction weakens β-amyloid clearance and autophagy in hippocampal neuronal cells, leading to learning and memory impairments.^110^ Endoplasmic reticulum (ER) stress promotes neuroinflammation, activates the NF-κB pathway, and contributes to cognitive decline.^111^ Furthermore, diabetes disrupts brain iron homeostasis, leading to neurotoxicity through inflammation, increased BBB permeability, altered iron ion redistribution, and impaired iron metabolism.^112^ Iron-chelating agents, such as desferrioxamine, represent potential therapeutic approaches for DE.^113^

Peripheral artery disease (PAD), characterized by restricted blood flow due to arterial stenosis or obstruction of arteries, results in tissue ischemia. Intermittent claudication, which presents as lower limb lameness after walking a certain distance and is relieved by a short rest, is a hallmark symptom of PAD.^114^ The disease pattern differs between diabetic and nondiabetic individuals. In diabetes-associated PAD, stenotic lesions predominantly affect distal arteries, such as the popliteal artery and the anterior tibial, posterior tibial and peroneal arteries, in contrast to the more proximal lesions seen in nondiabetic individuals.^115,116^ This distal involvement limits the development of collateral vessels and reduces revascularization options.^115^ Hyperglycemia induces vascular calcification via AGE accumulation, leading to hydroxyapatite deposits in both the intimal and medial layers, characteristic of atherosclerotic plaques and medial arterial calcification.^117^

The pathophysiological mechanisms underlying diabetes-related CVD involve endothelial dysfunction, oxidative stress, inflammatory cascade reactions, mitochondrial dysfunction, and ER stress, which form complex molecular networks. Future therapeutic strategies should focus on precision medicine guided by multiomics approaches and novel mitochondrial-targeted interventions, offering promising avenues to transform the management of diabetes-related CVD.

### Diabetic retinopathy (DR)

DR, affecting 34.6% of individuals with diabetes, is a leading cause of blindness.^118,119^ The pathogenesis of DR is complex, with emerging evidence highlighting the role of premature senescence in retinal cells and the secretion of inflammatory cytokines that exacerbate disease progression through paracrine senescence and pathological angiogenesis.^120,121^ Elevated blood glucose levels target ECs, leading to vascular injury. The loss of cell‒cell junctions between adjacent ECs and EC apoptosis are key drivers of acellular capillary formation and internal blood‒retinal barrier disruption.^22^ Hyperglycemia induces metabolic reprogramming in ECs, which is characterized by the accumulation of AGEs and the activation of the hexosamine, polyol, and PKC pathways. These changes promote oxidative stress, chronic inflammation, and premature EC senescence.^122^ Ferroptosis, an iron-dependent cell death mechanism characterized by lipid peroxide accumulation, has emerged as a novel therapeutic target in DR.^123^ TRIM46-induced ferroptosis in human retinal capillary endothelial cells involves glutathione peroxidase 4 (GPX4) ubiquitination and degradation, which are related to iron metabolism and DR pathology.^124^ Furthermore, multiple modes of cell death, including apoptosis, necroptosis, pyroptosis,^125^ and parthanatos,^126^ contribute to retinal ECs loss in DR.^127,128^

Advances in multiomics and artificial intelligence (AI) have facilitated noninvasive, high-resolution assessments of DR at the cellular level.^129^ Multiomics analyses have revealed metabolic shifts in retinal microglia,^130^ including a bias for glycolysis and reduced tricarboxylic acid cycle activity in diabetic models.^131^ A novel microglial subpopulation, termed immune microglia, shows immunoregulatory features with upregulation of the mitogen-activated protein kinase (MAPK), JAK/STAT, and IL-17 signaling pathways.^132^ The shared molecular features between renal mesangial cells and retinal pericytes, which are regulated by chemokines, further highlight common mechanisms in diabetes-related organ damage, as revealed through scRNA sequencing.^133^ Microglia‒endothelial interactions under hyperglycemic conditions are pivotal in DR progression. Hyperglycemia-induced EC secretion of colony-stimulating factor 1 activates microglia via CSF1R-mediated MAPK signaling, driving inflammation and angiogenesis. Necroptotic microglia expressing receptor-interacting protein 3 and mixed lineage kinase domain-like exacerbate retinal neovascularization by releasing fibroblast growth factor 2, which stimulates ECs.^132,134^ Moreover, neutrophil extracellular traps containing neutrophil elastase and DNA‒histone complexes induce oxidative stress, cellular senescence, apoptosis, and BRB disruption, further contributing to vascular dysfunction.^135^

High-throughput molecular profiling has established a gene expression atlas for retinal cells under hyperglycemic conditions, identifying novel cell subtypes involved in DR pathogenesis.^136^ scRNA-seq has identified insulin-like growth factor 1 (IGF-1) and secreted phosphoprotein 1 (Spp1)-expressing microglia as key sources of the proinflammatory cytokines IL-1β and TNF.^131,137^ Consistent with these findings, elevated vitreous Igf1 and Spp1 levels have been observed in DR patients compared with non-DR individuals.^138^ Pathological neovascularization, driven by VEGF and hypoxia-induced EC activation, is a hallmark of advanced DR. The discovery of G protein subunit alpha i2 (Gαi2) as a downstream mediator of VEGF signaling highlights its role in retinal angiogenesis via nuclear factor of activated T cells activation. These fragile neovessels are prone to rupture, leading to vision-threatening complications such as vitreous hemorrhage and tractional retinal detachment.^139^

Targeting microglial activation, ferroptosis, and EC–microglia crosstalk presents promising therapeutic opportunities. Integrating advanced molecular profiling and multiomics analyses offers a comprehensive understanding of DR pathogenesis, paving the way for innovative interventions to mitigate disease progression.

### Diabetic hepatopathy (DH)

The liver plays a pivotal role in glucose metabolism and insulin signaling, and its dysfunction exacerbates diabetes-related complications. Several key comorbidities, such as nonalcoholic fatty liver disease, are associated bidirectionally with T2D,^140^ which shares similar risk factors and pathophysiological mechanisms with DH. A hallmark of DH is hepatocellular lipid accumulation, or steatosis, resulting from insulin resistance. In this state, excess glucose is diverted into fatty acid synthesis via de novo lipogenesis, driven by the transcription factor sterol regulatory element binding protein-1c, which is upregulated under hyperglycemic conditions. Moreover, peroxisome proliferator-activated receptor-α, a nuclear receptor essential for fatty acid oxidation, is often downregulated, impairing lipid breakdown. These metabolic changes promote triglyceride storage within the liver, leading to lipid overload. Excessive lipid retention disrupts very low-density lipoprotein (VLDL) secretion, exacerbating hepatic steatosis and serving as a precursor to DH.^141^ Furthermore, emerging evidence suggests that the gut microbiota may influence the development of DH through the modulation of metabolism and inflammation. Dysbiosis increases intestinal permeability, enabling bacterial products to enter the bloodstream and trigger systemic inflammation, further compromising liver function.^142^ In summary, DH is characterized by insulin resistance, FFA accumulation, dysregulated lipid metabolism, and alterations in the gut microbiota. Understanding these mechanisms provides valuable insights into potential therapeutic strategies for managing liver complications associated with diabetes.

### Diabetic myopathy

Diabetic myopathy,^143^ a common complication of both T1D and T2D, involves the loss of muscle mass and function.^144^ Muscle tissues include cardiac, smooth, and skeletal muscle, and this discussion focuses on skeletal muscles. Metabolic disturbances caused by hyperglycemia adversely affect muscle function. Hyperglycemia activates the polyol pathway, increasing sorbitol and fructose production, which induces osmotic and oxidative stress in muscle cells. These stressors contribute to muscle cell damage and dysfunction.^145^ Diabetic myopathy is characterized by a metabolic shift from oxidative phosphorylation to glycolytic metabolism due to mitochondrial dysfunction. This shift reduces energy production and increases reliance on anaerobic pathways, leading to muscle fatigue and reduced force production.^146,147^ Furthermore, senescent muscle cells modify the ECM, creating an unfavorable environment for muscle regeneration. Changes in ECM composition and stiffness hinder satellite cell migration and differentiation into mature muscle fibers.^148^ The senescence-associated secretory phenotype, characterized by the release of proinflammatory mediators, exacerbates chronic inflammation in muscle tissue, further impairing muscle repair and regeneration.

### Diabetic peripheral neuropathy (DPN)

Despite advances in clinical care, DPN remains a prevalent complication of diabetes, with a lifetime incidence exceeding 50%.^149,150^ DPN alone accounts for over $10 billion in annual health-care costs and represents more than one-fourth of the total direct medical expenditures associated with diabetes.^151^ Among diabetic neuropathies, chronic diabetic sensorimotor peripheral neuropathy (DSPN) is the most common, accounting for approximately 75% of cases.^152^ Early symptoms typically involve burning, lancinating, tingling, shooting pain, and dysesthesias indicative of small myelinated nerve fiber involvement.^153^ However, large-fiber involvement is associated with numbness and the loss of protective sensation.^153,154^

The pathogenesis of DPN is driven primarily by metabolic disturbances characteristic of diabetes, including hyperglycemia and insulin resistance. Dysregulated lipid metabolism further complicates this condition. The accumulation of circulating lipids, particularly long-chain saturated fatty acids, impairs mitochondrial trafficking and increases lipotoxic acylcarnitines in Schwann cells (SCs), which may then be transferred to axons. Peripheral nerves develop insulin resistance, rendering insulin receptors on SCs and axons unresponsive. This insulin resistance disrupts SC and axon metabolism, diverting glycolytic intermediates into the polyol and hexosamine pathways.^155^ Hyperglycemia and dyslipidemia depolarize mitochondrial membranes, reducing ATP production and exacerbating an energy crisis while generating ROS.^156^ This combination of metabolic disturbances creates a vicious cycle of “bioenergetic failure,” resulting in distal-to-proximal nerve damage and producing the characteristic stocking‒glove pattern of DPN symptoms.^157^

Emerging research has focused on understanding DPN pathogenesis, including metabolism regulated through extracellular vesicles^158^ and the gut microbiome.^159^ Transplantation of the gut microbiota from DSPN patients (but not diabetes patients without neuropathy) into *db/db* mice treated with antibiotics resulted in exacerbated gut–barrier dysfunction, increased antigen load, systemic inflammation and aggravated peripheral neuropathy.^160^ Further genome-centric and guild-based approaches revealed a core microbiome cluster characterized by high butyrate production and reduced endotoxin synthesis, which was associated with the alleviation of DSPN.^160^ The underlying mechanism appears to involve immune infiltration^161^; for example, IgD-CD38-AC B cells mediate approximately 7.5% of the risk reduction for DPN via the thiazole biosynthesis I pathway in *E. coli*.^162^

### Diabetic foot ulcers (DFUs)

The global prevalence of DFUs is ~6.4% among patients with diabetes.^163^ Approximately 50–60% of patients with DFUs develop diabetic foot infections, and 15% ultimately undergo amputation.^164^ The pathogenesis of DFUs involves a complex interplay of vascular insufficiency, neuropathy, and microbial infections. PAD is a critical contributor to DFUs, impairing blood flow to the feet, which hinders wound healing and affects nearly half of all diabetic patients. Diabetic metabolic dysfunction, increased ROS, and chronic inflammation damage the vascular endothelium, promoting atherosclerosis through EC injury, vascular smooth muscle cell dysfunction, and platelet hyperactivity.^165^ Hyperglycemia, AGEs, acylcarnitine, and ox-LDL further exacerbate this condition by disrupting the integrity of nerve cells, leading to motor and sensory neuropathy. Sensory neuropathy reduces pain sensitivity, increasing the risk of unnoticed skin injuries and subsequent ulcers. Motor neuropathy presents as muscle atrophy, paralysis, and loss of reflexes, which result in structural changes such as Charcot foot and hammer toes. These structural abnormalities, combined with muscle weakness and imbalances, increase the risk of ulcer formation.^166^ Autonomic neuropathy contributes to vasomotor dysfunction, abnormal blood shunting in the skin vasculature, sweat gland dysfunction, and increased skin perfusion. These changes dry and weaken the skin, increasing the risk of ulceration. Diabetic foot infections are often polymicrobial, with common pathogens including *Staphylococcus aureus*, *Escherichia coli*, and *Pseudomonas* species.^167^ These pathogens exacerbate tissue destruction and inflammation, leading to chronic nonhealing wounds.^168^ Biofilm formation by these microorganisms protects against host immune responses and antimicrobial treatments, complicating infection eradication.^169^

### Molecular mediators that drive organ crosstalk

**Fig. 4:**
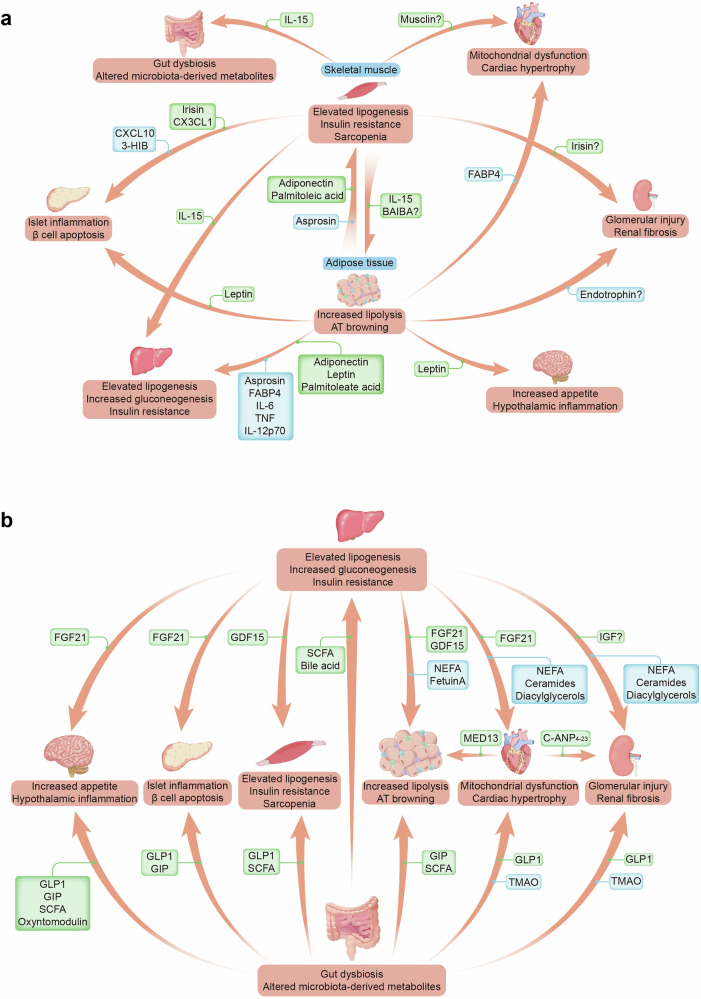
The role of mediators originating from skeletal muscle, adipose tissue, liver, intestine, heart, kidney and brain in inter-organ crosstalk. Factors that have protective or detrimental effects on each organ are shown in green and blue frames, respectively. The deleterious outcomes for each organ are listed under each organ, highlighted with orange frames. **a** Physical exercise triggers the release of skeletal muscle-derived myokines, while lipokines secreted from adipose tissue play important roles in the liver, heart, and kidney. IL-15 secreted from skeletal muscle and leptin from adipose tissue have beneficial effects on the intestine and brain, respectively. **b** Mediators secreted by the liver affect multiple tissues. Intestinal incretins (GLP-1, GIP) and short-chain fatty acids (SCFAs) from the microbiome exert beneficial effects on other organs, whereas trimethylamine-N-oxide (TMAO) plays a harmful role in diabetic retinopathy. Abbreviations: BAIBA β-aminoisobutyric acid, CXCL10 C-X-C motif ligand 10, CX3CL1 CX3C chemokine ligand 1, FABP4 fatty acid-binding protein 4, FGF21 fibroblast growth factor 21, IGF insulin-like growth factor, GDF15 growth differentiation factor 15, GIP gastric inhibitory polypeptide, GLP-1 glucagon-like peptide-1, HIB hydroxyisobutyrate, IL-15 Interleukin-15, IL-6 Interleukin-6, NEFA nonesterified fatty acid, PYY peptide YY, SCFAs short-chain fatty acids, TMAO trimethylamine-N-oxide, TNF tumor necrosis factor

Interorgan crosstalk among the kidney, heart, brain, adipose tissue, liver, skeletal muscle, pancreas, and intestine plays a pivotal role in the development of insulin resistance and β-cell dysfunction, which are central to the progression of diabetic complications. These organs communicate through various signaling pathways and factors, including adipokines, myokines, cytokines, hormones, and exosomes, mutually influencing each other’s functions and contributing to systemic metabolic dysfunction (Fig. 4).

#### Adipose and muscle crosstalk

Adipose tissue plays a key role in interorgan crosstalk by releasing numerous signals that communicate the body’s energy status to other tissues (Fig. 4a). Leptin—an adipose-derived hormone^170^—signals nutritional status, suppressing appetite and increasing energy expenditure via hypothalamic pro-opiomelanocortin neurons.^171^ In insulin resistance, elevated leptin levels reflect central leptin resistance, disrupting energy homeostasis and promoting hyperglycemia.^172^ Rodent^173^ and human studies^174^ reveal that leptin also modulates glucose and lipid metabolism through a brain–vagus–liver axis and hypothalamic–pituitary–adrenal signaling, contributing to adverse communication among adipose tissue, the hypothalamus, the liver and the pancreas, exacerbating T2D. Other critical mediators, including fatty acid-binding protein 4 (FABP4) and endotrophin, which are strongly associated with increased CVD risk in T2D patients, demonstrate significant crosstalk between adipose tissue, the liver, and the heart.^175,176^ Similarly, endotrophin is another adipokine that induces inflammation and fibrosis in adipose tissue. An animal model showed that blockade of endotrophin through neutralizing antibodies protects from renal fibrosis.^177^ Endotrophin might serve as a predictor of cardiovascular and renal morbidity, heart failure and overall mortality in individuals with T2D.^178^ Adipose tissue also releases mediators that counter diabetic complications through positive inter-organ crosstalk. Adipose tissue-derived adiponectin,^179^ a type of lipokine that includes palmitoleic acid (C16:1n7),^180^ contributes to interorgan communication with tissues such as the liver and muscle, with a potential role in ameliorating insulin resistance and type 2 diabetes in humans.

Myokines such as CXCL10, CX3CL1 (fractalkine), and follistatin influence the interaction between skeletal muscle and pancreatic β-cells.^181^ Evidence suggests that CXCL10 may have harmful effects by impairing insulin secretion and promoting β-cell apoptosis.^181^ In contrast, CX3CL1 appears to exert beneficial actions; chronic administration of a fractalkine analog in various rodent models of obesity has been shown to improve glucose tolerance and reduce β-cell apoptosis,^182^ underscoring its positive role in maintaining glucose homeostasis. However, myokines such as irisin, which increase energy metabolism by inducing browning of white adipose tissue, thereby promoting fatty acid oxidation and reducing hepatic gluconeogenesis,^183^ could inhibit the progression of diabetic complications by promoting beneficial interorgan crosstalk. Diabetic mouse models have shown that irisin alleviates glomerular injury and albuminuria.^184^ Moreover, exercise-induced exercise, such as 3-hydroxyisobutyrate, facilitates fatty acid accumulation and impairs insulin signaling in the pancreas and liver by reducing AKT phosphorylation-mediated pathways.^185,186^ β-Aminoisobutyric acid (BAIBA), a muscle-derived metabolite, supports energy metabolism by stimulating fatty acid oxidation and suppressing hepatic gluconeogenesis, acting as a protective factor against insulin resistance.^187^

Adipose and muscle-derived cytokines play pivotal roles in interorgan metabolic regulation. IL-15 enhances insulin sensitivity, promotes lipid oxidation, and activates the PPAR-δ pathway in muscle, liver, and fat, improving glucose homeostasis and reducing inflammation.^188^ Conversely, asprosin, interleukin-6 (IL-6), TNF-α, and IL-12p70, which are predominantly secreted by visceral adipose tissue,^189^ impair insulin signaling in muscle and liver and drive non-alcoholic fatty liver disease^190^ via pro-inflammatory mechanisms. This cytokine imbalance fosters deleterious adipose–muscle–liver–gut crosstalk, accelerating T2D progression.

The crosstalk among adipose tissue, skeletal muscle and other organs in diabetes is complex and bidirectional. Protective adipokines, altered lipokines, myokines, and metabolites from skeletal muscle-mediated communication can regulate metabolic homeostasis and mitigate the systemic effects of diabetes.

#### Liver as a metabolic signaling hub

The liver serves as a central endocrine and metabolic hub, coordinating interorgan communication to maintain energy homeostasis. It integrates signals from the gastrointestinal tract and adipose tissue, playing a vital role in regulating glucose and lipid metabolism in T2D. A key aspect of this crosstalk is the liver’s secretion of hepatokines, such as fibroblast growth factor 21 (FGF21) and growth differentiation factor 15 (GDF15), which increase insulin sensitivity,^191^ promote mitochondrial integrity in cardiomyocytes via the AMPK/FOXO3/SIRT3 signaling axis,^192^ and suppress renal fibrosis.^193,194^ Fetuin-A inhibits glucose-stimulated insulin secretion and, in conjunction with non-esterified fatty acids (NEFAs), activates Toll-like receptor 4-mediated proinflammatory pathways in adipocytes and macrophages.^195^ GDF15 regulates weight and glucose metabolism by suppressing caloric intake and reducing adaptive thermogenesis through its receptor GFRAL in the neurons of the area postrema and nucleus of the solitary tract.^196^ GDF15 further promotes fatty acid oxidation and lipid metabolism in skeletal muscle and adipose tissue, facilitating beneficial weight loss and glycemic improvements.^196^ Another critical hepatokine is IGF-1, which serves as a neurotrophic factor, assisting in nerve regeneration in sensory and motor neurons.^197^ However, IGF-1 plays dual roles in the kidney, supporting cell survival, whereas excessive signaling may promote fibrosis and podocyte injury.^198,199^

NEFA and lipid intermediates such as palmitate (C16:0), ceramides, and diacylglycerols released from the liver significantly impact the kidney and heart, particularly in conditions such as DKD^200^ and CVD.^201^ These lipid intermediates especially accumulate in renal proximal tubular cells, where increased lipid uptake exacerbates tubulointerstitial fibrosis and glomerulosclerosis, leading to progressive renal dysfunction.^202^ In the heart, excessive lipid deposition and the activity of lipid intermediates drive myocardial lipotoxicity, atherosclerosis, and diabetic cardiomyopathy.^203^ The liver–kidney–heart axis highlights the systemic impact of lipid-mediated signaling, which is associated with metabolic dysregulation, inflammation and insulin resistance, ultimately exacerbating both renal and cardiac pathologies.

Overall, the liver serves as a central endocrine and metabolic organ, coordinating extensive crosstalk between multiple systems. Through the secretion of hepatokines, bile acids, and metabolic signals, the liver links the gut, adipose tissue, muscle, and brain to maintain energy homeostasis. Disruptions in this communication, as observed in obesity and T2D, underscore the pivotal role of the liver in the pathophysiology of metabolic diseases. Targeting liver-mediated interorgan signaling represents a promising therapeutic strategy for mitigating metabolic dysfunction and improving systemic health.

#### Gastrointestinal‒endocrine crosstalk

The gastrointestinal tract serves as a major neuroendocrine hub, communicating with distant organs via intricate hormonal and neural signaling networks^204^ (Fig. 4b). A key mechanism of gastrointestinal communication is mediated by incretins, such as GLP-1 and GIP, which stimulate glucose-dependent insulin secretion after meals, facilitating entero-insular crosstalk.^205^ GLP-1 receptor expression in tissues such as the heart, kidneys, and immune cells highlights its systemic benefits, including cardiorenal protection and anti-inflammatory effects.^205–207^ Beyond this, GLP-1 exerts pleiotropic effects on multiple organs, including those involved in appetite control, whereas GIP directly influences metabolic processes in the endocrine pancreas and adipose tissue.^206^

The gut microbiota further influences neuroendocrine crosstalk due to reduced diversity in the gut microbiota, characterized by reduced abundance of *Faecalibacterium prausnitzii, Roseburia, Dialister, Flavonifractor, Alistipes, Haemophilus*, and *Akkermansia muciniphila*, along with an increase in *Lactobacillus, Streptococcus, Escherichia, Veillonella*, and *Collinsella*.^208^ Dysbiosis is implicated in diabetic complications, including CKD, CVD and retinopathy in diabetes.^209^ Dysbiosis, alterations in the composition of the gut microbiota characterized by perturbed eubiosis of the *Bacteroidetes* and *Firmicutes* phyla, impair intestinal barrier function, finally allowing lipopolysaccharides and other microbial products to enter the bloodstream, triggering systemic inflammation.^210^ Byproducts of the microbiota, such as trimethylamine N-oxide (TMAO) derived from dietary choline metabolism, have also been linked with DR, greater numbers of CVD events, and worse renal outcomes.^211–213^ However, short-chain fatty acids (SCFAs), including butyrate and propionate, derived from dietary fiber fermentation^207,214^ increase insulin sensitivity and energy metabolism by stimulating peptide YY and GLP-1 release and influencing hepatic function.^215^ Hormonal signaling from the GI tract also modulates adipose tissue and brain function. Ghrelin, produced by the stomach, stimulates appetite and regulates energy balance, whereas hormones such as peptide YY and oxyntomodulin suppress appetite and influence feeding behavior via neuroendocrine crosstalk with the brain, particularly through the hypothalamus.^216^ SCFAs, particularly acetate, activate the parasympathetic nervous system, which modulates ghrelin and insulin secretion,^217^ forming a complex feedback loop involving the gut, brain, liver and adipose tissue. Under pathological conditions, dysregulated SCFA production impairs protein synthesis, contributing to sarcopenia and chronic inflammation and exacerbating muscle loss and metabolic dysfunction.^218^

The gastrointestinal tract engages in extensive interorgan crosstalk, and therapeutic strategies targeting these pathways, including incretin-based therapies, DPP4 inhibitors, and microbiota modulation, such as fecal microbiome transplantation (FMT),^219^ offer promising approaches for improving insulin sensitivity and appetite regulation and mitigating diabetic complications. Understanding these complex interactions will advance the development of integrated treatments for T2D and its associated disorders.

#### Kidney‒heart axis in crosstalk

The kidney contributes to interorgan crosstalk through the secretion of hormones and proteins such as erythropoietin, renin,^220,221^ and Klotho,^222,223^ which have significant effects on the heart, muscle and adipose tissue. Klotho is predominantly expressed in the kidneys and is involved in promoting antioxidant defense functions by increasing the expression of superoxide dismutase, thus reducing the levels of ROS and preventing oxidative damage in the kidneys and heart.^224–226^ Additionally, Klotho enhances insulin sensitivity in peripheral tissues, including muscle and adipose tissue, and has neuroprotective functions through the modulation of neuronal signaling pathways.^222,223^ The protective effects of Klotho extend to the liver, where it contributes to the regulation of glucose and lipid metabolism and protects against liver fibrosis and steatosis.^227^

Cardiac crosstalk with the liver represents a vital axis of interorgan communication, primarily mediated through cardiomyokines such as natriuretic peptides, and secretory phospholipase A2 (sPLA2) regulates energy balance, lipid metabolism, inflammation and glucose homeostasis in diabetes.^228^ In addition to regulating sodium and volume homeostasis, atrial natriuretic peptide functions as an endocrine factor in the heart–liver axis by activating cGMP-protein kinase G-AKT-GSK3 signaling, leading to the regulation of liver glycogen metabolism.^216^ SPLA2, another cardiomyokine, can increase hepatic triglyceride levels and affect VLDL secretion, contributing to nonalcoholic fatty liver disease (NAFLD) and nonalcoholic steatohepatitis (NASH).^229^ C-Atrial natriuretic peptide (ANP)_4-23_, an agonist of natriuretic peptide receptor-C (NPR-C), reduces renal fibrosis by attenuating mineralocorticoid receptor (MR) activation and oxidative stress while modulating the Akt and Erk1/2 signaling pathways.^230^ Other heart-derived mediators, such as MED13, play a role in pathological adipocyte hypertrophy, with reduced MED13 expression observed in individuals with obesity and diabetes. Cardiac-specific deletion of MED13 increases susceptibility to obesity, whereas its overexpression promotes a lean phenotype.^228^ This complex cardiohepatic and heart‑to‑adipose crosstalk underscores the critical role of cardiomyokines in modulating metabolic hemostasis and systemic health.

DPD triggered by shared metabolic dysregulation and amplified through intersecting pathophysiological pathways constitutes a systemic vascular catastrophe traversing the entire circulatory continuum. It ultimately converges to produce severe multiorgan complications affecting the cardiac, cerebrovascular, renal, retinal, and peripheral vascular systems.^231^ Emerging biomarkers further highlight the complexity of interorgan crosstalk in diabetes. FGF21, which is primarily secreted by the liver in response to oxidative and endoplasmic reticulum stress, not only predicts DKD progression^232^ but also holds promise as a novel marker for NAFLD.^233^ Conversely, bone-derived FGF23 reflects mineral–metabolism disturbances and independently predicts incident DKD, adverse cardiovascular events and limb outcomes in diabetic individuals with peripheral arterial disease.^234,235^ Future research should aim to develop integrative, multiomic panels that combine hormonal, lipidomic, and genetic markers to generate dynamic “crosstalk signatures” that are predictive of organ-specific and systemic complications.

## Prevention of diabetic complications

### Maintaining a healthy lifestyle

The implementation of lifestyle changes to prevent complications of diabetes can yield substantial cost–benefit effects. Individuals who are overweight or obese are at increased risk of developing diabetes and should thus focus on behavioral changes that contribute to a healthy lifestyle.^236^ Lifestyle factors, including nutritional therapy, especially the Mediterranean diet,^237^ physical activity,^238–240^ smoking cessation,^241^ and quality sleep, are essential for preventing diabetes and its complications. Sleep and circadian rhythm disturbances are strongly linked to the development and poor outcomes of diabetes.^242^ Irregular sleep patterns, such as short or long durations, poor quality, or a late chronotype, are associated with increased insulin resistance and poor health outcomes.^243^ A U-shaped relationship exists between sleep duration and T2D risk, with 7–8 h of sleep per night corresponding to the lowest risk.^244^ Sleep duration variability is further associated with increased risks of CVD,^245^ DR and DKD.^246,247^ Sleep disorders are particularly prevalent among individuals with T2D,^248^ with those experiencing sleep disturbances for 15 or more days a month being at greater risk of complications.^249^ Interventions such as light therapy, sleep improvement strategies, and melatonin supplementation can help regulate circadian rhythms, potentially benefiting diabetes management and reducing complications, including DR.^250–253^ Therefore, effective diabetic complication management requires a holistic approach that integrates healthy lifestyle practices with advanced technologies, such as wearable health devices, which offer promising avenues for transforming diabetes care and improving patient outcomes.

### Controlling glycemia, blood pressure, and lipids

Achieving normoglycemia is essential for managing diabetes and preventing complications.^254^ While strict glycemic control effectively reduces microvascular complications,^255,256^ its impact on CVD risk is unclear and may increase adverse events in some populations.^257,258^ Dyslipidemia, commonly observed in T2D, is characterized by elevated triglycerides and low HDL cholesterol. Lipid-lowering therapies such as statins and PCSK9 inhibitors, particularly angiopoietin-like 3 antibodies and antisense oligonucleotide therapy, significantly reduce cardiovascular risk^76,259,260^ and provide additional benefits beyond cholesterol reduction, including anti-inflammatory and endothelial protective effects.^261^ Statins also have renoprotective effects by reducing albuminuria and preserving the glomerular filtration rate.^262^ Hemodynamic factors, such as the RAAS, significantly contribute to diabetic complications. As first-line therapies, RAAS inhibitors (RAASis) offer renal function preservation and cardiovascular protection with minimal side effects.^263–265^ Suggested screening strategies for various types of diabetic complications are detailed in Table 1.

**Table 1:** Screening for diabetic complications

Screening guidelines	Screening parameters	
Diabetic kidney disease		
Annually in T1D; with duration of ≥5 years in T2D	Urinary albumin (e.g., spot UACR), and eGFR^442^	
Diabetes-related cardiovascular disease		
In asymptomatic individuals, routine screening for coronary artery disease is not recommended.	-	
Atypical cardiac symptoms; signs or symptoms of associated vascular disease, including carotid bruits, transient ischemic attack, stroke, claudication, or peripheral arterial disease; or electrocardiogram abnormalities (e.g., Q waves).	Consider investigations for coronary artery disease^132^	
Adults with diabetes are at increased risk for the development of asymptomatic cardiac structural or functional abnormalities (stage B heart failure) or symptomatic (stage C) heart failure.	Measuring a natriuretic peptide (BNP or NT-proBNP)^132^	
In asymptomatic individuals with diabetes and abnormal natriuretic peptide levels.	Echocardiography^132^	
In asymptomatic individuals with diabetes and age ≥50 years, microvascular disease in any location, or foot complications or any end-organ damage from diabetes.	Ankle-brachial index testing^132^	
Peripheral arterial disease		
Diabetes duration ≥10 years	Ankle-brachial index testing, lower-extremity pulses, capillary refill time, rubor on dependency, pallor on elevation, and venous filling time^132,443^	
Diabetic retinopathy		
Within 5 years after the onset of T1D; At the time of the T2D diagnosis	Initial dilated and comprehensive eye examination; retinal photography with remote reading or the use of U.S. Food and Drug Administration–approved artificial intelligence^443^	
Diabetic hepatopathy		
Adults with T2D or prediabetes;		FIB-4 (derived from age, ALT, AST, and platelets)^355^
Adults with T2D or prediabetes with an indeterminate or high FIB-4		Liver stiffness measurement with transient elastography, the blood biomarker enhanced liver fibrosis.
Diabetic myopathy		
All elderly patients with diabetes		Questionnaire: SARC-F, SARC‑CalF; Imaging techniques: MRI, CT, BIA, and DXA; Anthropometric measurement techniques: MUAC, skinfold thickness, and calf circumference; Muscle Strength Measurement: handgrip strength and the chair stand test; Physical Performance Measurements: SPPB, SCPT^444–446^
Diabetic peripheral neuropathy		
5 years after the diagnosis of T1D; At diagnosis of T2D and at least annually thereafter		Small-fiber function: pinprick and temperature sensation. Large-fiber function: lower-extremity reflexes, vibration perception using a 128-Hz tuning fork, and 10-g monofilament. Protective sensation:10-g monofilament. Electrophysiological testing when necessary. Screening questionnaire: NSS, NSI, DNS, NDS, MNSIQ, MDNS, CSS, mTCNS^154,443,447–452^
Diabetic foot ulcers		
Annually in all diabetes		Inspection of the skin, assessment of foot deformities, neurological assessment (10-g monofilament testing with at least one other assessment: pinprick, temperature, or vibration), and vascular assessment, including pulses in the legs and feet.^443^

*T1D* type 1 diabetes, *T2D* type 2 diabetes, *UACR* urinary albumin-to-creatinine ratio, *eGFR* estimated glomerular filtration rate, *BNP* B-type natriuretic peptide, *NT-proBNP* N-terminal pro-BNP, *FIB-4* fibrosis-4 index, *ALT* alanine aminotransferase, *AST* aspartate aminotransferase, *SARC-F* strength assistance in walking rise from a chair climb stairs, *SARC‑CalF* strength‑assistance in walking‑rise from a chair‑climb stairs‑falls‑calf circumference questionnaire, *MRI* magnetic resonance imaging, *CT* computed tomography, *BIA* bioimpedance analysis, *DXA* dual-energy X-ray absorptiometry, *MUAC* mid-upper arm circumference, *SPPB* Short Physical Performance Battery, *SCPT* Stair Climb Power Test, *NSS* Neurological Symptom Score, *NSI* Neuropathy Screening Instrument, *DNS* Diabetic Neuropathy Score, *NDS* Neuropathy Disability Score, *MNSIQ* Michigan Neuropathy Screening Instrument Questionnaire, *MDNS* Michigan Diabetic Neuropathy Score, *CSS* Toronto Clinical Scoring System, *mTCNS* Modified Toronto Clinical Neuropathy Score

### Digital diabetes prevention program

Digital diabetes prevention programs, such as the National Health Service (NHS) Digital Stream and Omada Health’s model, have demonstrated efficacy in preventing T2D, particularly among high-risk individuals. The NHS Diabetes Prevention Program (DDP) uses digital tools, including apps, wearable devices, and virtual health coaches, to facilitate lifestyle modifications that delay or prevent diabetes onset and its complications.^266^ Ryan Batten et al. found that DDP is effective at preventing type 2 diabetes through a significant reduction in body weight and increase of physical activity.^267^ These programs allow for remote personalized care, addressing barriers such as travel limitations and time constraints while maintaining effective diabetes prevention outcomes.

## Treatment strategies for diabetic complications

### Multisystem effects of novel drugs in diabetic complications

Cardiovascular–kidney–metabolic syndrome (CKM), a multisystem disorder that is particularly prevalent in individuals with diabetes,^268^ highlights the interconnected risk factors and the need for integrated management strategies. Recent advances in pharmacotherapy highlight the potential of novel agents to concurrently target multiple diabetic complications, reshaping therapeutic paradigms. This section synthesizes evidence on the systemic effects of metformin, sodium‒glucose cotransporter 2 inhibitors (SGLT-2Is), GLP-1RAs, nonsteroidal mineralocorticoid receptor antagonists (ns-MRAs), and dual incretin agonists across organ systems.

Metformin, a first-line antidiabetic drug, exhibits modest cardioprotective effects and reduces mortality in T2D,^72^ although its impact on the incidence of major adverse cardiovascular events (MACE) remains neutral.^269,270^ Its utility is limited in patients with advanced CKD owing to safety concerns.^271^ Emerging preclinical evidence suggests the potential of metformin in ameliorating NAFLD. In *db/db* mice, metformin has been shown to reduce iron accumulation and lipid-related ROS production in the liver, thereby mitigating liver injury.^136^ Mechanistically, metformin modulates the expression of genes^272^ associated with hepatic inflammation and fibrosis,^273^ consequently improving hepatic stiffness and slowing NAFLD progression,^274^ although clinical translation requires further validation.

SGLT-2 is expressed in multiple organs beyond the kidneys and heart, including the brain, liver, and retina.^275,276^ SGLT-2Is reduce MACE (primarily by reducing cardiovascular death),^277^ hospitalizations due to heart failure,^278^ and CKD progression^278^ (including significant reductions in albuminuria and delayed eGFR decline) in various populations, irrespective of baseline glycemic status. Animal studies suggest that SGLT-2Is provide neuroprotective effects by mitigating neuroinflammation, increasing cerebral glucose metabolism, and limiting amyloid protein aggregation.^279–281^ A systematic review of RCTs suggested that SGLT-2Is are associated with a lower occurrence of neuropathy events (SGLT-2I: 3.81% vs control: 4.18%).^282^ Clinical observational studies have also shown that SGLT-2Is lower the risk of cognitive decline (montreal cognitive assessment scores improved by 2.5),^283^ dementia, and Parkinson’s disease (approximately 20% reduction).^284^ Furthermore, electronic medical records studies have shown that SGLT-2 slows DR progression^285^ and decreases reliance on anti-VEGF therapies,^286^ probably by enhancing retinal fuel metabolism, reducing oxidative stress, and improving retinal neurovascular coupling.^276,287^ Moreover, SGLT-2Is have shown potential for treating NASH and NAFLD through the inhibition of hepatocellular glucose uptake and subsequent modulation of pathways associated with oxidative stress, inflammation, autophagy, and apoptosis.^288^ Observational studies have linked SGLT-2Is to NAFLD regression and reduced liver-related outcomes in patients with comorbid T2D and NAFLD.^289^ An RCT of empagliflozin also confirmed these findings, showing significant reductions in hepatic fat content by 2.49% after 52 weeks.^290^ Large-scale phase III trials (e.g., NCT06519448 and NCT06218342) are now underway to verify these benefits and refine the role of SGLT-2Is in NAFLD management. Despite these advances, the effects of SGLT-2Is on diabetic myopathy^291–293^ and DFUs^294–297^ remain unclear, necessitating further research.

GLP-1R, a key member of the G protein-coupled receptor family, is ubiquitously expressed on the surfaces of various cells, such as pancreatic β-cells, hepatocytes, and cells in the cardiovascular and neural systems, and has significant therapeutic potential for multiple diseases.^298^ GLP-1RAs and dual incretin receptor agonists are strongly recommended for the treatment of diabetes, particularly in overweight or obese individuals.^299^ Notably, semaglutide and tirzepatide (a dual GIP and GLP-1RA) can lead to effective weight loss, marking a new era in weight management. Additionally, these drugs offer both cardiovascular and renal benefits, strengthening their use in diabetes management. The findings of meta-analyses and clinical trials indicate that semaglutide not only reduces the risk of MACE and heart failure^300,301^ but also improves renal outcomes in obese patients with or without diabetes.^302,303^ The SURPASS-4 trial^304^ revealed that tirzepatide significantly reduced composite kidney endpoints by 40% and improved the annual eGFR decline by 2.2 mL/min per 1.73 m^2^ per year. Further analyses revealed that tirzepatide dose-dependently decreased the levels of atherogenic lipoproteins, such as apoC-III and apoB, which are major cardiovascular risk factors.^305^ CagriSema (a combination of semaglutide and the long-acting amylin analog cagrilintide) has completed phase II clinical trials^306^ and has been shown to be superior to both tirzepatide and semaglutide in terms of promoting weight loss (−14.03 kg, −8.47 kg, and −3.13 kg, respectively, for 3 months of treatment),^307^ making it one of the most noteworthy drugs of 2025.

Apart from reducing appetite and delaying gastric emptying to lose weight, GLP-1RAs exhibit potential benefits in modulating innate immune responses and inhibiting β-cell apoptosis.^308^ Ongoing phase III clinical trials of semaglutide have aimed to further elucidate the efficacy and safety of these drugs in the T1D population (NCT05819138). In addition, GLP-1RAs exert neuroprotective effects by restoring brain energy metabolism, enhancing BBB integrity, and reducing neurovascular inflammation, oxidative stress, and apoptosis.^309–312^ These mechanisms highlight the potential of GLP-1RAs in treating DE and diabetic neuropathy. Small-scale RCTs and observational studies have shown that GLP-1RAs enhance impaired odor-induced brain activation^312^ and improve the size of the tibial nerve and sural sensory nerve conduction amplitude in T2D.^313^ Meta-analyses have associated GLP-1RAs with lower risks of dementia,^314^ cognitive decline,^315^ and pain disorders.^316^ In addition to their neuroprotective effects, GLP-1RAs indirectly improve hepatic insulin resistance, lipotoxicity, and inflammation,^317^ with promising results in improving liver histology.^318–320^ A phase 2 RCT revealed that 0.4 mg semaglutide induced 42% greater NASH resolution than did placebo.^320^ In 2023, the American Association for the Study of Liver Diseases guidance recommended the use of semaglutide for managing metabolic dysfunction-associated steatohepatitis in patients with T2D or obesity, even without FDA approval.^321^ Additionally, dual incretin receptor agonists, including tirzepatide and GLP-1/glucagon receptor coagonists such as efinopegdutide, pemvidutide, and cotadutide, have shown promise in reducing liver fat and resolving fibrosis in NAFLD and related conditions.^322–326^ Among them, the 15 mg tirzepatide group achieved 52% NASH resolution compared with the placebo group.^326^ However, weight loss induced by GLP-1RA has been associated with reductions in muscle mass, which can lead to sarcopenia and frailty.^327^ To mitigate these effects, personalized resistance exercise is recommended to preserve muscle mass during GLP-1RA therapy. Ongoing pharmacologic strategies aim to maintain or improve muscle mass during GLP-1RA therapy.^328^

Finerenone, a ns-MRA, has demonstrated significant efficacy in reducing the risk of clinically important cardiovascular and kidney outcomes in patients with T2D and DKD.^329^ Ongoing clinical trials are also investigating its effects on T1D and DKD (NCT05901831, Phase III)^330^ Beyond its renal and cardiovascular advantages, finerenone has shown promise in treating DR. A subset of participants from the FIDELIO-DKD and FIGARO-DKD trials underwent routine ophthalmological evaluations, revealing a lower incidence of vision-threatening complications in the finerenone group (3.7% [5/134]) than in the control group (6.4% [7/110]).^331^ While these findings suggest that finerenone may delay the progression of nonproliferative DR in T2D patients with DKD, the lack of randomization and the limited number of endpoint events restrict the strength of these conclusions. Preclinical investigations have indicated that finerenone reduces retinal inflammation, vascular leakage, and microglial density, thereby supporting its potential therapeutic role in DR management.^332,333^ Furthermore, on the basis primarily of preclinical evidence, MRAs have been shown to confer protection against cognitive decline in hypertensive conditions.^334,335^ Additionally, MRAs may improve muscle function, reduce degradation and inflammation, and mitigate fibrosis in dystrophic muscles.^336^

These findings underscore the multifaceted benefits of novel drugs in treating diabetic complications, highlighting their potential as comprehensive therapeutic agents in diabetes management. Recent multicenter RCTs have demonstrated that SGLT-2Is,^278,337–339^ GLP-1RAs,^340^ and ns-MRAs^329,341,342^ offer significant kidney and cardiovascular benefits, regardless of baseline albuminuria, eGFR, or diabetes status.^76,343,344^ Initial therapy with finerenone plus SGLT-2Is led to a greater reduction in the urinary albumin-to-creatinine ratio than either treatment alone did,^345^ which was consistent with a meta-analysis indicating that the combination of RAASis, SGLT-2Is, and ns-MRAs—the so-called “renal triple therapy”—synergistically reduces cardiorenal events with minimal risk of hyperkalemia.^346,347^ These findings mark the onset of a new treatment paradigm for CKM disorders and the use of drug combination therapies to significantly lower multisystemic risks in patients with T2D by targeting multiple mechanisms.^348^

**Fig. 5:**
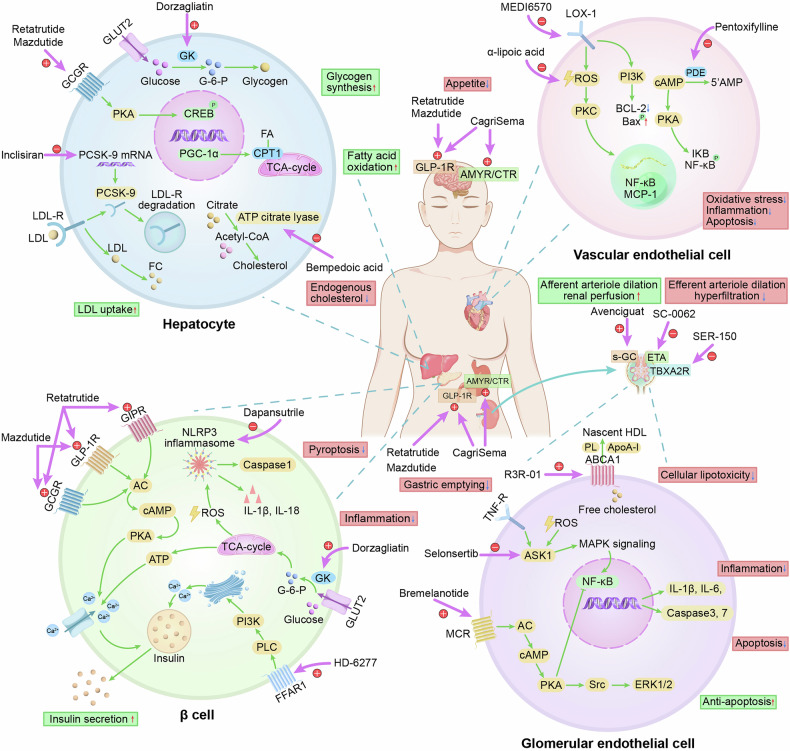
Promising molecular targeted drugs for treating diabetic complications. This figure illustrates the selected promising molecularly targeted drugs for diabetes, diabetic kidney disease, and diabetes-related cardiovascular diseases. GLP-1R, GIPR, and GCGR agonists enhance insulin secretion in β-cells via the cAMP/PKA pathway. GCGR agonists further activate CREB to promote hepatic fatty acid oxidation. GLP-1R and AMYR/CTR agonists regulate appetite and slow gastric emptying. Mazdutide, Retatrutide, and CagriSema, owing to their pharmacological structures or combinatorial formulations, activate multiple receptors simultaneously. Dorzagliatin enhances glucose-stimulated insulin secretion and hepatic glycogen synthesis by activating glucokinase. HD-6277 targets FFAR1 to activate the PLC pathway, increasing insulin release. Dapansutrile inhibits the NLRP3 inflammasome, reducing β-cell inflammation and pyroptosis to preserve function. In DKD, avenciguat activates sGC, dilating afferent arterioles and improving renal perfusion. SER-150 and SC-0062 inhibit TBXA2R and ETA receptors, dilating efferent arterioles to reduce glomerular hyperfiltration. Selonsertib and bremelanotide mitigate glomerular endothelial inflammation and apoptosis by targeting ASK1 and melanocortin receptors, respectively. R3R-01 promotes cholesterol efflux via ABCA1 to generate nascent HDL, reducing cellular lipotoxicity. Inclisiran, a siRNA, degrades PCSK9 mRNA, preventing LDLR degradation and enhancing hepatic LDL clearance. Bempedoic acid inhibits ATP citrate lyase in the liver, reducing endogenous cholesterol synthesis. MEDI6570 blocks LOX-1 to attenuate vascular endothelial inflammation and apoptosis. Furthermore, pentoxifylline inhibits PDE, increasing cAMP levels to exert anti-inflammatory effects. Alpha-lipoic acid, an antioxidant, scavenges ROS to reduce oxidative stress, inflammation, and apoptosis. Abbreviations: ABCA1 ATP-binding cassette transporter A1, AC adenylate cyclase, AMYR amylin receptor, ApoA-I apolipoprotein A-I, ASK1 apoptosis signal-regulating kinase 1, BCL-2 B-cell lymphoma 2, CREB cAMP response element-binding protein, CPT1 carnitine palmitoyltransferase 1, CTR calcitonin receptor, DKD diabetic kidney disease, ERK extracellular signal-regulated kinase, ETA endothelin A receptor, FA fatty acids, FC free cholesterol, FFAR1 free fatty acid receptor 1, GCGR glucagon receptor, GIPR glucose-dependent insulinotropic polypeptide receptor, GK glucokinase, GLP-1R glucagon-like peptide-1 receptor, GLUT2 glucose transporter 2, HDL high-density lipoprotein, LDL low-density lipoprotein, LDLR low-density lipoprotein receptor, LOX-1 lectin-like oxidized low-density lipoprotein receptor-1, MAPK mitogen-activated protein kinase, MCP-1 monocyte chemoattractant protein-1, MCR melanocortin receptor, NF-κB nuclear factor kappa-light-chain-enhancer of activated B cells, NLRP3 NACHT LRR and PYD domains-containing protein 3, PCSK9 proprotein convertase subtilisin/kexin type 9, PDE phosphodiesterase, PGC-1α peroxisome proliferator-activated receptor gamma coactivator 1-alpha, PI3K phosphoinositide 3-kinase, PKA protein kinase A, PKC protein kinase C, PL phospholipase, PLC phospholipase C, ROS reactive oxygen species, sGC soluble guanylate cyclase, siRNA small interfering RNA, Src proto-oncogene tyrosine-protein kinase, TBXA2R thromboxane A_2_ receptor, TCA tricarboxylic acid, TNF-R tumor necrosis factor receptor

Emerging novel drugs are redefining therapeutic approaches by addressing multiple diabetic complications concurrently. Targeting NLRP3 inflammasome activation has direct translational relevance: the small-molecule inhibitor MCC950 ameliorates albuminuria, glomerulosclerosis, and podocyte injury in *db/db* mice by blocking caspase-1/IL-1β maturation.^349^ EndMT drives renal fibrosis in DKD, and overexpressed bone morphogenetic protein-7 prevents EndMT and extracellular matrix deposition in diabetic rodent models.^350^ Likewise, FGF21 analogs reduce urinary albumin excretion, mesangial expansion, and oxidative stress in db/db mice, linking their metabolic and antifibrotic actions to a promising DKD therapy.^351^ Details of other promising candidates under active clinical investigation in the past five years are presented in Table 2, with a more comprehensive list of drugs provided in Table S1. Figure 5 illustrates the mechanisms by which the drugs act on their targets, primarily for the treatment of diabetes, DKD, and diabetes-related cardiovascular disease.

**Table 2:** Promising drug under active development for diabetic complications over the past 5 years

Drug name	Target	Clinical trials’ number (Phase)	Condition	Outcomes	Year
Dorzagliatin	Glucokinase activators	NCT03141073 (phase III)	T2D	Change from baseline in HbA1c	2022
Mazdutide	GLP-1R and GCGR agonists	NCT05606913 (phase III, no results); NCT05607680 (phase III, no results)	T2D; Obesity	HbA1c change from baseline at week 28; Percent change from baseline in body weight	2022
Retatrutide	GIPR, GLP-1R, and GCGR agonists	NCT06354660 (phase III, no results); NCT06662383 (phase III, no results)	T2D; Obesity	Change from baseline in HbA1c; Percent change from baseline in body weight	2024
CagriSema	AMYR/CTR, and GLP-1R agonists	NCT06534411 (phase III, no results); NCT06780449 (phase III, no results); NCT05669755 (phase III, no results); NCT06131372 (phase II, no results)	T2D; Obesity; CVD; DKD	Change in HbA1c; Relative change in body weight; Time to first occurrence of MACE; Change in UACR	2024
Dapansutrile	NLRP3 inhibitors	NCT06047262 (Phase II, no result);	T2D	Change in serum HbA1c	2023
HD-6277	FFAR1 agonists	NCT06647550 (Phase II, no result);	T2D	Change in HbA1c from baseline at week 12	2024
SER-150	TBXA2R antagonists	NCT04881123 (phase II/III, no results)	DKD	Change of UACR of > 30% from baseline to day 168	2021
Selonsertib	ASK1 inhibitors	NCT04026165 (phase IIb)	DKD	eGFR slopes	2024
Bremelanotide	Melanocortin receptor agonists	NCT05709444 (phase IIb, no results)	DKD	Ratio achieving a 50% reduction in UP/Cr	2023
Avenciguat	Soluble guanylate cyclase activator	NCT04750577 (phase II)	DKD	Change in UACR in 10-hour urine	2024
R3R-01	ABCA1 inducer	NCT06600412 (phase II, no results)	DKD	Change in UACR from baseline to 12 weeks	2024
SC-0062	ETA inhibitor	NCT05687890 (phase II, no results)	DKD	Percentage change from baseline in UACR	2022
Inclisiran	SiRNA target PCSK9	NCT03399370 (phase III); NCT03400800 (phase III); NCT03397121 (phase III)	ASCVD or elevated cholesterol in diabetes	Change in LDL-c	2023
Bempedoic acid	ATP citrate lyase inhibitor	NCT02993406 (phase III)	CVD in diabetes	A four-component composite of major adverse cardiovascular events	2024
MEDI6570	Anti-LOX-1	NCT05912218 (phase II, no results)	T2D and peripheral arterial disease	Lower limb atheroma plaque inflammation	2023
Pentoxifylline	Phosphodiesterase inhibitor	EudraCT #2009-016595-77 (not applicable)	Atherosclerosis in T2D	Rotid intima-media thickness and ABI	2024
Alpha-lipoic acid	Anti-ROS	NCT06056687 (not applicable, no result)	Ischemic cardiomyopathy in diabetes	Change in inflammatory and fibrosis markers levels and LV echocardiography improvements	2023
OCS-01	GR agonists	NCT06172257 (phase III, no results)	DME	Change in BCVA	2023
Aflibercept	Anti-VEGF	NCT04429503 (phase II/III)	DME	Change in BCVA	2024
Restoret	FZD4, LRP5 agonists	NCT06571045 (phase II/III, no results)	DME	Change in BCVA	2024
RC-28	Anti-VEGF/FGF-2	NCT05885503 (phase III, no results)	DME	Change in BCVA	2023
Vamikibart	Anti-IL-6	NCT05151731 (phase II, no result)	DME	Change in BCVA	2021
BI-764524	Anti-SEMA3A	NCT06321302 (phase II, no result)	Non-proliferative diabetic retinopathy	Occurrence of a more than 2-step improvement in DRSS level	2024
Lanifibranor	PPARRα/β/γ activator	NCT03459079 (phase II)	T2D and MASLD	Change in intrahepatic triglycerides	2025
PF-06835919	Ketohexokinase inhibitor	NCT03969719 (phase II)	T2D and NAFLD	Percent change from baseline in whole liver fat and HbA1c at week 16	2022
Tipelukast	LTD4R, LTC4R and TBXA2R antagonists, PDE3, PDE4, and ALOX5 inhibitors	NCT05464784 (phase II, no results)	T2D and NAFLD	Mean change in controlled attenuation parameter score by sound-based elastography	2022
MET-409	FXR agonists	NCT04702490 (phase II, no results)	T2D and NASH	Chang in magnetic resonance Imaging-derived proton density fat fraction	2021
Tirzepatide	GIPR and GLP-1R agonists	NCT05751720 (phase Ⅰ/II, no results)	T2D and NAFLD	Change in liver stiffness	2023
Acetyllevocarnitine Hydrochloride	Acetylcholine modulators	NCT05319275 (phase III)	DPN	Modified Toronto Clinical Neuropathy Score	2024
HSK16149	Voltage-gated calcium channel α2δ subunit ligand	NCT04647773 (phase III)	DPN	Average daily pain score	2024
Oxybutynin	Muscarinic receptor antagonists	NCT03050827 (phase III)	DPN	Intraepidermal nerve fiber density	2024
Suzetrigine	SCN10A (Nav1.8) channel blockers	NCT06696443 (phase III, no results)	DPN	Change from baseline in SF-36v2-PCS Score	2024
ISC-17536	TRPA1 inhibitors	NCT01726413 (phase II)	DPN	Mean 24-hour average pain intensity score based on an 11-point pain intensity numeric rating scale	2022
LX-9211	AAK1 inhibitors	NCT04455633 (phase II)	DPN	Average daily pain score	2024
Topical Esmolol Hydrochloride	β1-adrenergic receptor inhibitors	NCT03998436 (phase III)	DFU	Proportion of wound closure	2023
ENERGI-F703	AMPK activators	NCT05930210 (phase III, no results)	DFU	The ulcer complete closure rate	2023
TP-102	A phage cocktail targeting multidrug-resistant bacteria and biofilms	NCT05948592 (phase II, no result)	DFU	DFUWI score and percentage of patients who achieve a 50% reduction in wound surface area	2023
Pravibismane	Multidrug-resistant bacteria and biofilms	NCT05174806 (phase II, no result)	DFU	Proportion of subjects with complete wound closure	2021
ILP-100	A lactic acid bacteria express CXCL-12	NCT05608187 (phase II, no result)	DFU	Percent of wound area reduction	2022

Note: The clinical trial information for all listed drugs can be searched on clinicaltrials.gov or pubmed.ncbi.nlm.nih.gov using their clinical trial numbers. Detailed references can be found in Table S4

*AAK1* adapter protein-2-associated kinase 1, *ABCA1* ATP-binding cassette transporter A1, *ABI* ankle-brachial index, *ALOX5* Arachidonate 5-Lipoxygenase, *AMPK* AMP-activated protein kinase, *AMYR* amylin receptor, *ASCVD* atherosclerotic cardiovascular disease, *ASK1* apoptosis signal-regulating kinase 1, *BCVA* best-corrected visual acuity, *CTR* calcitonin receptor, *CVD* cardiovascular disease, *CXCL-12* chemokine (C-X-C motif) ligand 12, *DFU* diabetic foot ulcers, *DFUWI* diabetic foot ulcer with infection, *DKD* diabetic kidney disease, *DME* diabetic macular edema, *DPN* diabetic peripheral neuropathy, *DRSS* diabetic retinopathy severity scale, *ETA* endothelin A receptor, *FFAR1* free fatty acid receptor 1, *FGF-2* fibroblast growth factor-2, *FXR* farnesoid X receptor, *FZD4* frizzled class receptor 4, *GCGR* glucagon receptor, *GIPR* gastric inhibitory polypeptide receptor, *GLP-1R* glucagon-like peptide-1 receptor, *IL-6* interleukin-6, *LDL-c* low-density lipoprotein cholesterol, *LOX-1* lectin-like oxidized low-density lipoprotein receptor 1, *LRP5* LDL receptor related protein 5, *LTC4R* leukotriene C4 receptor, *LTD4R* leukotriene D4 receptor, *LV* left ventricle, *MACE* major adverse cardiovascular events, *MASH* metabolic dysfunction-associated steatohepatitis, *MASLD* metabolic dysfunction-associated steatotic liver disease, *NAFLD* nonalcoholic fatty liver disease, *NASH* nonalcoholic steatohepatitis, *NLRP3* NACHT LRR and PYD domains-containing protein 3, *PCSK9* proprotein convertase subtilisin/kexin type 9, *PDE* Phosphodiesterase, *PPAR* peroxisome proliferator-activated receptor, *ROS* reactive oxygen species, *SCN10A* sodium channel protein type 10 subunit alpha, *SEMA3A* semaphorin-3A, *SF-36v2-PCS* 36-item short-form health status physical component summary, *T2D* type 2 diabetes, *TBXA2R* thromboxane A2 receptor, *TRPA1* transient receptor potential ankyrin 1, *UACR* urinary albumin-to-creatinine ratio, *VEGF* vascular endothelial growth factor

### Stem cells and stem cell-derived exosomes: regenerative and immunomodulatory potential for treating diabetic complications

Mesenchymal stem cells (MSCs) are promising therapeutic candidates for regenerative medicine. Sources of MSCs, such as placenta,^352^ adipose tissue,^353^ and human umbilical cord,^354^ provide versatile platforms for therapeutic applications. After intravenous administration, MSCs initially localize to the lungs and liver before homing to target organs such as the kidneys, where they exert antidiabetic effects by mitigating inflammation and fibrosis, partly via the autophagy-mediated Sirtuin 1 (SIRT1)/Forkhead Box O1 pathway.^355^ Clinical evidence supports their safety and efficacy. Table S2 shows the therapeutic and immunomodulatory effects of stem cells or their exosomes on diabetic complications, involving the regulation of macrophage polarization, the inflammatory balance, and Tregs. A phase 1b/2a trial demonstrated that a single infusion of allogeneic MSCs slows the decrease in the eGFR in patients with progressive DKD.^356^ Synergistic effects are observed when MSCs are combined with conventional therapies, such as GLP-1RA exenatide^357^ or SGLT-2I, such as empagliflozin,^358^ which improve mitochondrial autophagy, podocyte protection, and renal function in diabetic rat models. These combinations exhibit synergistic anti-inflammatory effects, suppress DNA damage, and regulate cytokines.^359^ Furthermore, genetic engineering of MSCs to overexpress angiotensin-converting enzyme-2 ameliorates DKD by modulating the TGF-β/Smad signaling pathway and reducing glomerular fibrosis.^360^ Advances in bioengineering techniques, including 3D encapsulation,^361^ hydrogels,^362^ and nanoparticles,^363^ have increased MSC survival, differentiation, and therapeutic potential. However, challenges such as chromosomal abnormalities, potential tumorigenesis, and suboptimal integration in diabetic microenvironments remain.^364^

MSC-derived exosomes (MSC-Exos) provide a safer, cell-free approach that minimizes the risks of immune rejection and tumorigenesis.^365^ These nanovesicles deliver bioactive molecules, including proteins, lipids, and nucleic acids, to mediate intercellular communication and modulate recipient cell behavior.^366^ Stem cells and stem cell-derived exosomes have shown immunomodulatory effects on DKD, diabetic cardiomyopathy, DR, and DFU, involving the regulation of macrophage polarization, the inflammatory balance, and Tregs (Table S2). Additionally, exosomal miRNAs from adipose-derived MSCs enhance autophagy flux and alleviate podocyte injury by suppressing mTOR signaling, leading to reductions in proteinuria and serum creatinine levels in DKD patients.^367^ Exosomes derived from bone marrow MSCs,^368^ human umbilical cord MSCs,^369^ and urine-derived stem cells^370^ show similar promise in DKD management.

In diabetic cardiomyopathy, MSC-Exos improve cardiac dysfunction by alleviating the inflammation associated with the TAK1-pJNK-NFKB pathway.^371^ In diabetic myocardial injury, MSC-Exos mitigate fibrosis and myocardial damage by inhibiting the TGF-β1/Smad2 signaling pathway.^372^ MSC-Exos reduce retinal vascular endothelial injury and inflammatory cytokine production in DR by downregulating the expression of markers such as high mobility group box 1, NLRP3, and NF-κB/P65.^373^ Moreover, MSC-Exos deliver miR-222 to retinal cells, regulating signal transducer and activator of transcription 5 signaling, inhibiting neovascularization, and promoting retinal regeneration in advanced DR.^374^ MSC-Exos also promote M2 macrophage polarization, suppress inflammation and enhance wound healing in diabetic ulcers.^375,376^ Advances in engineering and molecular profiling continue to improve their efficacy, paving the way for innovative clinical applications. Further research is essential to fully realize their potential and ensure safe, effective deployment in managing diabetes and its complications.

The development of smart drug delivery systems for treating diabetes, incorporating glucose-sensing components such as glucose-binding proteins, glucose oxidase, and phenylboronic acid, together with advanced carriers such as hydrogels, microgels, and nanoparticles, has ensured precise, safe, and efficient insulin delivery.^377^ The use of esterified collagen hydrogels can increase the differentiation and functionality of insulin-producing cells derived from induced pluripotent stem cells (iPSCs).^378^ Chen et al. successfully encapsulated vascularized islets composed of iPSC-derived β-like cells and microvascular fragments via three-dimensional (3D) printing combined with hydrogels, ensuring high survival of islet cells and low immunogenicity.^379^ Coculture of iPSC-derived β-cells with endothelial cells and their integration into a bioengineered vascular system enabled the creation of a functional, sustainable, humanized endocrine organ, with a controlled in vitro insulin-secreting phenotype and effective in vivo function.^380^ This biomimetic pancreas, composed of β and α cells derived from human iPSCs and GLP-1 analog-loaded glucose-responsive nanoparticles, was found to increase survival rates in diabetic mice,^381^ suggesting promising prospects for future diabetes treatment. Furthermore, novel drug delivery systems can substantially increase stem cell viability and therapeutic efficacy for diabetic complications by providing 3D microenvironments that support cell migration, proliferation and differentiation.^382^ These systems enable precise cell targeting and sustained therapeutic release, as demonstrated by Wang et al. in treating DKD, using placental mesenchymal stem cells with guided nanoparticles.^383^ Furthermore, the integration of biomaterials and genetic engineering techniques is expected to further augment the therapeutic potential of stem cells.^384^ In DR, nanotechnology offers transformative solutions through controlled, targeted therapies.^385,386^ Notably, Lee et al. developed a dopamine-functionalized gellan gum hydrogel that enhanced retinal pigment epithelium function by increasing the expression of vision-related genes.^387^ Hydrogel-based and scaffold-based delivery platforms show particular promise for DFUs, as they effectively promote wound healing and skin regeneration.^388,389^ As these technologies continue to evolve, careful evaluation of their biocompatibility and long-term safety will be essential for maximizing their clinical benefits.

### Gut flora: regulating inflammation and metabolic health

The gut microbiota plays a critical role in maintaining homeostasis and metabolic health, with disruptions in its composition associated with the progression of diabetic complications.^390,391^ Emerging therapies, including prebiotics, probiotics, and FMT, aim to modulate the gut microbiota and its metabolites, illustrating the potential to mitigate complications associated with diabetes.^209^

Previous studies have reported that probiotic products and FMT can improve renal parameters, such as plasma urea nitrogen and serum creatinine levels, in patients with CKD.^392,393^ A novel prebiotic, the graminan-type fructan from *Achyranthes bidentata*, has the potential to prevent DKD. This prebiotic alleviates kidney injury by promoting the production of SCFAs and modulating the gut microbiota composition, increasing the abundance of *Bacteroides* while decreasing the abundance of *Rikenella* and *Alistipes* in DKD mice.^394^ Similarly, enriched seafood sticks containing postbiotic and bioactive compounds have shown efficacy in lowering cardiometabolic risk factors, including HOMA-IR and postprandial triglyceride concentrations, which is partially attributed to changes in the composition of the gut microbiota.^395^ In diabetic mouse models, *Lactobacillus paracasei* has been shown to reduce retinal inflammation, gliosis, neuronal cell death, and vascular capillary loss, thereby mitigating DR.^396^ In a double-blind, placebo-controlled RCT, FMT from healthy donors significantly alleviated DSPN in recipients. Compared with the placebo group (10 patients), those who received FMT (22 patients) presented enriched beneficial microbial guilds and suppressed harmful guilds.^160^ Moreover, intermittent fasting improved cognitive dysfunction in *db/db* mice by reconstructing the gut microbiota and altering microbial metabolites, likely *via* increased mitochondrial biogenesis and energy metabolism in the hippocampus.^397^ Adjunctive probiotic therapy also improves the therapeutic effects of conventional medications in managing T2D. By promoting SCFA-producing bacteria and modulating bile acid pathways, probiotics increase the efficacy of standard treatments.^398^

Current clinical data on targeting the gut microbiota for diabetic complications remain limited, with particular gaps in understanding the modulation of specific microbial compositions and their therapeutic efficacy. Advances in microbiome research may allow personalized interventions targeting dysbiosis patterns associated with specific diabetic complications.^399^ Furthermore, the interplay between gut microbiota-based therapies and conventional medications presents promising opportunities for integrated treatment strategies. The ongoing exploration of the role of the gut microbiota in systemic inflammation and metabolic health highlights its potential as a key therapeutic target in diabetes management.

### Traditional Chinese Medicine: synergistic approaches to integrative regulation and precision therapies

The long-term management of diabetes typically involves the lifelong use of antidiabetic medications, which often impose economic burdens and are associated with undesirable side effects, leading to poor adherence among patients.^400,401^ Compared with synthetic drugs, traditional Chinese medicine (TCM) has gained popularity as an alternative or complementary approach because of its perceived safety, efficacy, and holistic benefits compared to synthetic drugs (Table S3).

Resveratrol (RES), a naturally occurring phytoalexin found in cereals, fruits, vegetables, and plant-derived beverages such as tea and wine, has diverse biological activities, including antiobesity, antidiabetic, anticancer, anti-inflammatory, antioxidative, and cardiovascular-protective effects.^402^ Resveratrol protects the heart from I/R injury and cardiomyopathy through multiple mechanisms, such as scavenging free radicals, reducing myocardial oxygen demand, inhibiting inflammation-induced damage, inducing angiogenesis, improving mitochondrial function, and preventing cardiomyocyte apoptosis.^403^ A study integrating network pharmacology, molecular docking, and experimental validation revealed that RES can target the PPARA, SHBG, AKR1B1, PPARG, IGF1R, MMP9, AKT1, and INSR domains, acting as a therapeutic agent for DKD.^404^ In addition, resveratrol ameliorates diabetic retinopathy by preserving blood–retinal barrier integrity and suppressing inflammation and oxidative stress through AMPK activation, SIRT1 preservation, NF-κB inhibition, Nrf2/GPx4 pathway regulation.^405^

Berberine, a bioactive alkaloid derived from TCM herbs such as *Rhizoma Coptidis*, exerts anti-inflammatory, antioxidative, hepatoprotective, and anticancer effects.^406–408^ A phase 2 RCT demonstrated that berberine ursodeoxycholate significantly reduced liver fat content, improved glycemic control, lowered liver enzyme levels, and promoted weight loss in T2D patients with presumed NASH.^409^ In DKD, berberine inhibits podocyte apoptosis, ROS generation, and mitochondrial dysfunction.^410^ Berberine also alleviates DR by inhibiting insulin-induced activation of retinal ECs through the Akt/mTOR/HIF-1α/VEGF pathway^411^ and reduces DCM by suppressing IL-1β secretion and gasdermin D expression.^412^ Moreover, other TCM formulations, such as Rehmannia-6-based medicine^413^ and Astragalus,^414^ have shown promising effects. Both have been reported to stabilize the eGFR after 48 weeks in patients with T2D and DKD when used alongside standard care.

Despite its promise, the use of TCM for managing diabetic complications faces several challenges. Many current clinical studies on TCM interventions for diabetic complications fail to fully adhere to the principles required for high-quality RCTs, including multicenter collaboration, adequate sample size, randomization, blinding, Good Clinical Practice, and research ethics for human subjects, resulting in lower levels of evidence in these RCTs.^415^ Consequently, the U.S. FDA has not approved TCM interventions for treating diabetic complications.^416^ The complexity of TCM formulas, which are composed of multiple medicinal herbs with numerous uncharacterized components, impedes in-depth experimental investigations.^417^ Additionally, the characteristic pattern differentiation-based treatment approach inherently necessitates timely prescription adjustments, including modifications to formula composition and dosage, on the basis of patients’ evolving clinical manifestations across different treatment periods.^418^ Collectively, these factors critically limit the application of TCM in the management of diabetic complications. By addressing these challenges, TCM could provide a valuable, integrative approach to managing the complex pathogenesis of diabetes and its complications, offering a multicomponent, multitarget synergistic treatment paradigm.

### Beyond traditional care: the digital health paradigm shift in the management of diabetic complications

Digital health technologies (DHTs), particularly AI, transform diabetes care by addressing critical challenges in prevention, diagnosis, and management^171^ (Fig. 1). Traditional medical practices often encounter issues such as delayed diagnoses, insufficient healthcare resources, and the need for continuous self-management.^419,420^ AI, combined with wearables, mobile apps, and telemedicine, offers innovative solutions to these problems, improving efficiency and patient outcomes.^421^ AI algorithms have demonstrated significant potential in predicting diabetic complications, enabling targeted interventions for high-risk individuals.^422^ Advances include AI-powered diabetic retinopathy screening systems such as AEYE Health (AEYE Health Inc.), EyeArt (Eyenuk Inc.), and IDx-DR (IDx LLC), which facilitate early and accurate detection.^423,424^ The EyeArt system is a cloud-based automated AI eye screening technology designed to detect referable DR by automatically analyzing patients’ retinal images. An early version of the EyeArt system software (v1.2) demonstrated 90% sensitivity and 63.2% specificity on a data set of 40,542 images from 5084 patient visits.^425^ A recently reported real-world study involving over 100,000 consecutive visits by diabetic patients revealed that automated DR screening via the EyeArt system v2.0 achieved high screening sensitivity (91.3%) and specificity (91.1%).^424^ A separate independent study of the EyeArt system on more than 20,000 consecutive patient encounters revealed that the sensitivity and specificity were not affected by patient ethnicity, sex, or camera type.^426^ Additionally, the EyeArt system is a computerized, cost-effective, cloud-based AI medical device capable of screening approximately 100,000 patients in less than 45 h, whereas manual graders can evaluate only 8–12 patients per hour.^424^ However, the widespread adoption of AI-driven platforms such as the EyeArt system raises critical ethical concerns, particularly regarding data privacy and security.^427^ In addition, while cloud-based AI improves screening efficiency, it escalates the risk of unauthorized third-party data access.^428^ Similarly, AI has been applied in DKD screening and management, as shown by the Minuteful Kidney system. This system uses a step-by-step kit to detect kidney damage by identifying abnormalities in the UACR, allowing remote and accessible screening.^429,430^ Wearable technologies, such as Checkme Lite, employ AI algorithms to detect up to 45 types of abnormal electrocardiogram (ECG) events, offering rapid analysis, early warnings, and timely interventions. Innovations in diabetic foot care include assessments via thermography and smartphone imaging,^431,432^ whereas neuropathy screening benefits from AI integration in electronic health records and imaging techniques.^433–435^ Platforms such as NVIDIA Clara support AI-driven applications in imaging and drug discovery, enabling the development of 3D organ models, such as kidneys, to assess organ volume and enhance diagnostic precision.

Telemedicine has shown improved outcomes compared with traditional care, with evidence indicating better reductions in HbA1c through remote consultations.^436,437^ Mobile apps and smart devices further improve diabetes management by enabling patient education, continuous monitoring, and seamless data sharing between patients and healthcare providers.^438,439^ AI facilitates home-based monitoring, community screening programs, and hospital-based complication detection, paving the way for personalized treatment algorithms and integrated healthcare systems. These advancements promise to improve clinical outcomes while reducing healthcare costs, highlighting the transformative potential of DHTs in diabetes care. Nevertheless, the implementation of AI-driven diagnostic platforms such as EyeArt raises critical ethical considerations, particularly concerning data privacy and security vulnerabilities.^427^ Furthermore, while cloud-based deployment enhances screening accessibility, it concomitantly introduces risks of unauthorized third-party data access.^428^

## Conclusion and future perspectives

The mechanisms associated with diabetic complications involve complex interactions across multiple organs and systems. Although spatial multiomics and single-cell omics techniques can provide a deeper understanding of tissue and cellular heterogeneity, elucidation of the molecular and phenotypic heterogeneity in disease pathways underlying diabetic complications and the complex interplay of risk factors, such as obesity, aging, and inflammation, poses significant challenges to the discovery of biomarkers and the development of standardized therapeutic strategies. Machine learning models using these data sets show promise but still require validation in diverse cohorts.

MSCs and MSC-derived exosomes may represent a cutting-edge therapeutic approach for managing diabetic complications, offering regenerative benefits with reduced risks. Despite encouraging preclinical and early clinical results, several critical gaps must be addressed before MSC- and MSC-derived exosome-based therapies for diabetic complications can be developed. First, the long-term biosafety of MSC-derived exosomes is still undetermined. Rigorous toxicology assessments are needed to identify and eliminate off-target effects or harmful components, ensuring the safety of exosome-based therapies.^440^ Second, there is an urgent need to establish optimal dosing regimens, biodistribution patterns, and treatment schedules for exosome administration in diabetic complications. To date, most work has been limited to exosome administration in vitro, so comprehensive preclinical investigations including extended follow-up to evaluate both efficacy and safety in vivo are urgently needed. Although AI has been widely applied in the diagnosis, prognosis prediction, and personalized treatment of diabetic complications, its translation into healthcare demands additional scrutiny. AI systems depend heavily on the breadth and diversity of their training data sets, rendering models less reliable for populations underrepresented in the data and raising concerns about their generalizability and the introduction of bias.^441^ Furthermore, machine learning models limit clinicians’ ability to interpret decisions, detect errors, and build confidence in AI-driven recommendations. Nevertheless, integrating AI with bioinformatics may accelerate the elucidation of the underlying mechanisms of diabetic complications and drive the creation of personalized treatments through large-scale data analytics.

## Supplementary information

Supplementary Material_cleaned version
