# Supplementary material for: Diabetes and its complications: molecular mechanisms, prevention and treatment
Source: Signal Transduct Target Ther. 2026 Jan 19;11:22. doi: 10.1038/s41392-025-02401-w (PMC12812840; doi:10.1038/s41392-025-02401-w)
Supplement: Supplementary file 1 — Supplementary Material_cleaned version [file 41392_2025_2401_MOESM1_ESM.docx]

Supplementary Materials for

Diabetes and its complications: molecular mechanisms, prevention and treatment

Lijun Zhao1,2*, Jiamin Yuan1,2*, Qing Yang1,2*, Jing Ma1,2*, Fenghao Yang3*, Yutong Zou1,2, Ke Liu1,2, Fang Liu1,2＃

Correspondence to: liufangfh@163.com

**This PDF file includes:**

Tables S1 to Drugs for diabetic complications under active development.

Tables S2 to The therapeutic and immunomodulatory effects of stem cells or their exosomes on diabetic complications.

Tables S3 to Clinical trials of prescription formulas for diabetic complications.

Tables S4 to The references of clinical trials in Table 2.

Table S1. Drugs for diabetic complications under active development.

| **Drug name** | **Mechanism of Action** | **Phase** | **Condition** |  |
| --- | --- | --- | --- | --- |
| **Diabetes** | | | |  |
| CagriSema | AMYR/CTR, and GLP-1R Agonists | III III  III  II | T2D;  Obesity;  CVD; DKD |  |
| Dorzagliatin | Glucokinase Activators | III | T2D; |  |
| Mazdutide/IBI362 | GLP-1R and GCGR Agonists | III  III | T2D; Obesity |  |
| Retatrutide | GIPR, GLP1R, and GCGR Agonists | III  III | T2D;  Obesity |  |
| Volagidemab | Antibody antagonist to the human glucagon receptor | II I/II | T1D; T2D |  |
| Icovamenib | Menin/KMT2A Interaction Inhibitors | II | T1D |  |
| HD-6277 | FFAR1 Agonists | II | T2D |  |
| IONIS-GCGRRx | GCGR Expression Inhibitors（Antisense Therapy) | II | T2D |  |
| APL-18881 | FGF receptor1 Activators and GLP-1R Agonists | II | T2D |  |
| HR-17031 | GLP-1R Agonists, Insulin Mimetics, and Insulin Receptor Agonists | II | T2D |  |
| Linerixibat | SLC10A2 Inhibitors | II | T2D |  |
| Bezisterim | Insulin Sensitizers and NFKB Inhibitors | II | T2D |  |
| Dapansutrile | NLRP3 Inhibitors | II | T2D |  |
| CK-0045 | Class II Cytokine Receptors | II  II | T2D; Obesity |  |
| GLY-200 | Mucins | II  II | T2D; Obesity |  |
| S-723595 | ACC2 Inhibitors | II | T2D |  |
| Tesofensine/metoprolol Succinate | 5-HT Reuptake Inhibitors, ADRB1 Antagonists, Dopamine Reuptake Inhibitors, and Norepinephrine Reuptake Inhibitors | II  II | T2D; Hypothalamic obesity |  |
| Azemiglitazone Potassium | Insulin Sensitizers MPC Inhibitors | II  II | T2D; NASH |  |
| XEN-D0501 | TRPV1 Antagonists | II | T2D |  |
| GSK-1070806 | Anti-IL-18 | II | T2D |  |
| Pradigastat | DGAT-1 Inhibitors | II  II  II | T2D;  NAFLD;  Coronary artery disease |  |
| Isosteviol | Potassium Channel Activators | II | T2D |  |
| Tolimidone | Tyrosine-Protein Kinase Lyn Activators | II | T2D |  |
| **Diabetic kidney disease** | | | |  |
| SER-150 | TBXA2R Antagonists | II/III | DKD |  |
| Selonsertib | ASK1 | II | DKD |  |
| Avenciguat | Soluble guanylate cyclase | II | DKD |  |
| Bremelanotide acetate | MC3R and MC4R Agonists | II | DKD |  |
| SCO-792 | Enteropeptidase Inhibitors | II | DKD |  |
| Dimethylfumarate | Keap1 Ligands, NLRP3 Inhibitors, ME1 Inhibitors, NFKB Activation Inhibitors, and NFE2L2 Activators | II | DKD |  |
| Propagermanium | CCR2 Antagonists | II | DKD |  |
| Azeliragon | RAGE Receptor Antagonists | II | DKD |  |
| GLY-230 | AGE Inhibitors | II | DKD |  |
| Imarikiren Hydrochloride | Renin Inhibitors | II | DKD |  |
| Isuzinaxib Hydrochloride | NOX3 Inhibitors NADPH Oxidase Complex Inhibitors | II | DKD |  |
| MIB-626 | precursor of NAD+ | II | DKD |  |
| Praliciguat | sGC Activators | II | DKD |  |
| R3R-01 | ABCA1 Activators | II | DKD |  |
| Reflocibart | Anti-VEGF | II | DKD |  |
| SC-0062 | ETA Antagonists | II | DKD |  |
| TMX-049 | XDH Inhibitors | II | DKD |  |
| VPI-2690B | Anti-CD51/CD61 | II | DKD |  |
| Monlunabant/INV-202 | Cannabinoid CB1 Receptor Inverse Agonists | II | DKD |  |
| **Diabetes related cardiovascular disease** | | | |  |
| AT-001 | Aldose Reductase Inhibitors | III | Diabetic cardiomyopathies |  |
| Apabetalone | APOA1 Expression Enhancers and BRD2/3/4 Inhibitors | III | T2DM and coronary artery disease |  |
| Inclisiran | PCSK9 siRNA | III | ASCVD or elevated cholesterol in diabetes |  |
| Bempedoic Acid | ATP citrate lyase | III | CVD in diabetes |  |
| Ninerafaxstat Trihydrochloride | Fatty Acid Oxidation Inhibitors | II | Diabetic cardiopathy |  |
| MEDI6570 | LOX-1 | II | T2D and peripheral arterial disease |  |
| Pentoxifylline | Phosphodiesterase | not applicable | Atherosclerosis in T2D |  |
| Alpha-lipoic acid | ROS | not applicable | Ischemic Cardiomyopathy in diabetes |  |
| **Diabetic hepatopathy** | | | |  |
| Lanifibranor | PPAR Agonists | II | T2D and MASLD |  |
| PF-06835919 | ketohexokinase | II | T2D and NAFLD |  |
| Saroglitazar | PPARalpha Agonists | II | T2D and NASH |  |
| PXL065 | PPARgamma Agonists | II | T2D and NASH |  |
| Efinopegdutide | GLP1R and GCGR Agonists | II | T2D and NAFLD |  |
| Tipelukast/MN-001 | LTD4R, LTC4R and TBXA2R antagonists, PDE3, PDE4, and ALOX5 inhibitors | II | T2D and NAFLD |  |
| Tirzepatide | | GIPR and GLP-1R Agonists | I/II | T2D and NAFLD |
| **Diabetic retinopathy** | | | |  |
| Ruboxistaurin mesilate Hydrate | PKC beta Inhibitors | III | DR |  |
| RC-28 | Anti- VEGF/ FGF-2 | III | DME |  |
| OCS-01 | GR Agonists | III | DME |  |
| Restoret | FZD4 and LRP5 Agonists | II/III | DME |  |
| Aflibercept | Anti- VEGF | II/III | DME |  |
| Vamikibart | IL-6 | II | DME |  |
| ABBV-RGX-314 | Gene therapy comprising a recombinant AAV8 encoding an VEGF-directed antibody fragment | II | DR |  |
| Vorolanib | KDR, PDGFR and Tyrosine Kinase inhibitor | II | DME |  |
| Aganirsen | IRS1 Expression Inhibitors | II | DME |  |
| AVD-104 | An engineered glycan (sialic acid) nanoparticle molecule | II | DME |  |
| PER-001 | ETR Antagonists | II | NPDR |  |
| D-didesethyl sunitinib conjugate | Angiogenesis Inhibitors CSF1 receptor Inhibitors, KIT Inhibitors, PDGFRalpha Inhibitors, PDGFR beta Inhibitors, and VEGF receptor 1/2 Inhibitors | II | DME |  |
| BI-764524 | Anti- SEMA3A | II | NPDR |  |
| Foselutoclax | Bcl-2 Inhibitors | II | DME |  |
| AG-73305 | Angiopoietin-1 Receptor and VEGF | II | DME |  |
| INV-102 | TP53 Activators | II | DME |  |
| HCB-1019 | Gap junction Connexin and Gap Junction Protein | II | DR |  |
| THR-149 | Plasma Kallikrein Inhibitors | II | DME |  |
| ISTH-0036 | TGFB2 Expression Inhibitors | II | DME |  |
| Nesvategrast | Vitronectin and Integrin Receptor Antagonists | II | DME |  |
| BI-1026706 | Bradykinin B1 Receptor Antagonists | II | DME |  |
| Runcaciguat | sGC Activators | II | NPDR |  |
| Risuteganib | VLA-2 receptor and Integrin Receptor | II | DME |  |
| Lazucirnon | CCR3 Antagonists | II | NPDR |  |
| EG-Mirotin | Human ADAM-15 derived protein and Angiogenesis Inducers | II | DME |  |
| OPL-0401 | ROCK 1/2 Inhibitors | II | NPDR |  |
| Anti-PIGF | Anti-PGF | II | DME |  |
| APX-3330 | DNA-lyase and NFKB Inhibitors | II | DME |  |
| **Diabetic peripheral neuropathy** | | | |  |
| HSK16149 | Voltage-gated calcium channel α2δ subunit ligand | III | DPN |  |
| Oxybutynin | Muscarinic receptor | III | DPN |  |
| Acetyllevocarnitine Hydrochloride | L-carnitine | III | DPN |  |
| Suzetrigine | SCN10A (Nav1.8) Channel Blockers | III | DPN |  |
| Choline diepalrestat | Aldose Reductase Inhibitors | II/III | DPN |  |
| Cebranopadol Hemicitrate | Opioid Receptor and Nociceptin Receptor Agonists | II | DPN |  |
| Desmetramadol | Mu-Opioid Receptor Agonists and Norepinephrine Reuptake Inhibitors | II | DPN |  |
| Pilavapadin/LX-9211 | AAK1 Inhibitors | II | DPN |  |
| Mazisotine | SSTR4 Agonists | II | DPN |  |
| MEDI-7352 | NGF and TNF Receptors | II | DPN |  |
| Pirenzepine | Muscarinic M1 Receptor Antagonists | II | DPN |  |
| Cemdomespib | HSP90 Inhibitors | II | DPN |  |
| Ricolinostat | Histone Deacetylase 6 Inhibitors | II | DPN |  |
| CNV-2197944 | N-Type Calcium Channel (Ca(v) 2.2) Blockers | II | DPN |  |
| ISC-17536 | TRPA1 Antagonists | II | DPN |  |
| NRD.E1 | Lyn tyrosine kinase | II | DPN |  |
| Cibinetide | IRR Agonists | II | DPN |  |
| VX-993 | NaV1.8 sodium channel | II | DPN |  |
| **Diabetic foot ucler** | | | |  |
| DermaPro | Dichloric acid applied via a moist wound dressing | Pre-Registered | DFU |  |
| Gentamicin sulfat | 16S Ribosomal RNA (Bacterial) Inhibitors, 30S Ribosomal Subunit (Bacterial) Inhibitors, and Nonsense Mutation Suppressors | III | DFU |  |
| Donaperminogene Seltoplasmid | A DNA vector expressing two isoforms of HGF | III | DFU |  |
| ENERGI-F703 | AMPK Activators | III | DFU |  |
| SR-0379 | Angiogenesis Inducers | III | DFU |  |
| Anacaulase-bcdb | Enzymatic debriding agent | II | DFU |  |
| AUP-16 | A genetically engineered Lactococcus lactis expressing FGF2, IL4 and CSF1 | II | DFU |  |
| Human FGF1 | FGF1 | II | DFU |  |
| ILP-100 | A genetically engineered lactic acid bacteria that express the human chemokine CXCL-12 | II | DFU |  |
| TP-102 | A phage cocktail targeting strains of Pseudomonas aeruginosa, Staphylococcus aureus and Acinetobacter baumannii | II | DFU |  |
| Pravibismane | Multidrug-resistant bacteria and biofilms | II | DFU |  |

**Notes:** All the above data can be retrieved from the CDDI (https://www.cortellis.com/drugdiscovery/), PubMed (https://pubmed.ncbi.nlm.nih.gov/), or ClinicalTrials (https://clinicaltrials.gov/) databases by searching the drug name.

**Abbreviation:** AAK1, AP2-associated protein kinase 1; ABCA1, phospholipid-transporting ATPase; ACC2, acetyl-CoA carboxylase 2; ADAM15, disintegrin and metalloproteinase-15; ADRB, beta-adrenoceptor; AAV8, recombinant adeno-associated viral vector 8; AGE, advanced glycation end-product; ALOX5, polyunsaturated fatty acid 5-lipoxygenase; AMPK, AMP-activated protein kinase; AMYR, amylin receptor; APOA1, apolipoprotein A1; ASCVD, atherosclerotic cardiovascular disease; ASK1, apoptosis signal-regulating kinase 1; BD2, bromodomain-containing protein 2; Bcl-2, B-cell lymphoma 2; BRD, bromodomain-containing protein; CALCR, calcitonin receptor; CB1, cannabinoid receptor 1; CCR, C-C chemokine receptor; CSF1, macrophage colony-stimulating factor 1; CVD, cardiovascular disease; CXCL12, C-X-C motif chemokine ligand 12; DGAT1, diacylglycerol acyltransferase type 1; DFU, diabetic foot ulcers; DKD, diabetic kidney disease; DME, diabetic macular edema; DPN, diabetic peripheral neuropathy; DR, diabetic retinopathy; ETA, endothelin A receptor; ETR, endothelin receptor; FGF, fibroblast growth factor; FFAR1, free fatty acid receptor 1; FZD4, frizzled-4; GIPR, gastric inhibitory polypeptide receptor; GCGR, glucagon receptor; GLP-1R, glucagon-like peptide 1 receptor; GR, glucocorticoid receptor; HGF, hepatocyte growth factor; HSP90, heat shock protein 90; IL4, interleukin-4; IL6, interleukin-6; IL18, interleukin-18; IRR, innate repair receptor; IRS1, insulin receptor substrate 1; KDR, kinase insert domain receptor; LOX1, lectin-like oxidized low-density lipoprotein receptor-1; LRP5, low-density lipoprotein receptor-related protein 5; LTC4R, leukotriene C4 receptor; LTD4R, leukotriene D4 receptor; MASH, metabolic dysfunction-associated steatohepatitis; MASLD, metabolic dysfunction-associated steatotic liver disease; MC3R, melanocortin MC3 receptor; MC4R, melanocortin MC4 receptor; NAFLD, non-alcoholic fatty liver disease; NASH, non-alcoholic steatohepatitis; KMT2A, lysine methyltransferase 2A; NAD, nicotinamide; NFKB, nuclear factor kappa B; NGF, nerve growth factor; NLRP3, NACHT, LRR and PYD domains-containing protein 3; NOX3, NADPH oxidase 3; NPDR, non-proliferative diabetic retinopathy; PCSK9, proprotein convertase subtilisin/kexin type 9; PDE, phosphodiesterase; PDGFR, platelet-derived growth factor receptor; PGF, placental growth factor; PKC, protein kinase C; PPAR, peroxisome proliferator-activated receptors; RAGE, advanced glycation end-product receptor; ROCK, rho-associated protein kinase; ROS, reactive oxygen species; sGC, soluble guanylate cyclase; SCN10A, sodium channel protein type 10 subunit alpha; SEMA3A, semaphorin-3A; 5-HT, 5-Hydroxytryptamine; SLC10A2, solute carrier family 10 member 2; SSTR4, somatostatin receptor type 4; TBXA2R, thromboxane A2 receptor; T1D, type 1 diabetes; T2D, type 2 diabetes; TGFB2, transforming growth factor beta 2; TNF, tumor necrosis factor; TP53, cellular tumor antigen p53; TRPA1, transient receptor potential cation channel subfamily A member 1; TRPV1, transient receptor potential cation channel subfamily V member 1; VEGF, vascular endothelial growth factor; VLA, Integrin alpha; XDH, xanthine dehydrogenase.

Table S2. The therapeutic and immunomodulatory effects of stem cells or their exosomes on diabetic complications.

| **Disease** | **Model** | Stem Cells/ exosomes | **Effects on Diabetic Complications** | **Signaling pathway** | **Immunomodulatory Effects** |
| --- | --- | --- | --- | --- | --- |
| Diabetic kidney disease | Streptozotocin (STZ)-induced diabetic rats | Human placenta-derived mesenchymal stem cells (MSCs) | Improved renal function and alleviated pathological changes, including tubular vacuolar degeneration, mesangial matrix expansion, and glomerular filtration barrier injury. | Upregulating PD-1 and downregulating programmed death-ligand 1 (PD-L1) pathway | Increased the proportion of Treg cells and reduced the proportion of Th17 cells;  Reduce pro-inflammatory cytokines such as IL-17A and IL-1β in the kidney and blood^1^ |
| Diabetic kidney disease | STZ-induced diabetic rats | Human umbilical cord MSCs-derived miR-146a-5p | Decrease in Scr, serum BUN and 24h total urinary protein;  Reduce systemic and local renal inflammation | Inhibiting TRAF6/signal transducer and STAT1 signaling pathway | Facilitate M2 macrophage polarization (shifted macrophage polarization from a pro-inflammatory M1 to an anti-inflammatory M2 phenotype) ^2^ |
| Diabetic kidney disease | STZ-induced diabetic mice | MSCs | increased the survival rate and alleviated the renal injuries | Improving transcription factor EB-mediated autophagy activity in Mφ | Promote M2 polarization; inhibited F4/80+ Mφ infiltration in renal tissue; downregulated the expressions of TNF-α, MCP-1, and IL1β, but upregulated the expression of IL-10^3^ |
| Diabetic kidney disease | STZ-induced diabetic rats | Human umbilical cord-MSCs | Ameliorated functional parameters;  Reduce renal vacuole degeneration, inflammatory cell infiltration, and renal interstitial fibrosis | TGF-β signaling pathway | Reduced the levels of pro-inflammatory cytokines (IL-6, IL-1β, and TNF-α) and pro-fibrotic factor (TGF-β) in the kidney and blood; Secreted large amounts of growth factors^4^ |
| Diabetic kidney disease | db/db mice | Human umbilical cord-MSCs | Attenuate podocyte injury under high glucose | Restrained the TLR signaling pathway | Reduce the expression of IL-6, IL-β, TNF-α, MCP-1^5^ |
| Diabetic kidney disease | STZ with HFD-induced diabetic rats | Adipose-derived MSCs | Reduce Scr, BUN, and the urinary ACR;  Ameliorate renal fibrosis | Not mentioned | Reduce the number of iNOS(+) M1 macrophages and restored the number of CD163(+) M2 macrophages; Inhibit pro-inflammatory cytokines IL-6, IL-1β, and TNF-α expression; increase anti-inflammatory cytokine IL-10 ^6^ |
| Diabetic kidney disease | db/db mice | Human umbilical cord MSCs-derived exosomes | Reduce 24-h urinary albumin excretion rate, and Scr; Improve mesangial area dilation, and diminished tubular vacuolar degeneration | Not mentioned | Enhance M2 macrophages polarization. ^7^ |
| diabetic cardiomyopathy | STZ with high-glucose, HFD-induced diabetic mice | MSCs | Improve LVEF, FS, E′/A′ ratio;  Reverse Cardiac hypertrophy and the degree of myocardial fibrosis | miR-223-3p/NLRP3 pathway | Suppress the activation of NLRP3 and reduce the release of inflammatory factors (such as IL-1β and ROS);  Downregulating the expression of pyroptosis-related proteins including NLRP3, Caspase-1, and GSDMD^8^ |
| Diabetes-associated atherosclerosis | HFD and STZ ApoE-/- mouse model | BMSC-EVs | Reduce blood glucose, LDL levels, and aortic plaque areas; increase collagen content | Inhibit AMPK/mTOR signaling pathway | Inhibiting vascular macrophage proliferation, migration, and foam cell formation; Inhibit macrophage polarization^9^ |
| Diabetic retinopathy | db/db mice | hESC derived MSCs | Enhance impaired retinal electroretinography functions (including amplitudes of a-, b-wave and oscillatory potentials);  Reducing non-perfusion area and increasing vascular area density in the inner retina | Not mentioned | Increase anti-inflammatory cytokine IL-10; Decreased pro-inflammatory cytokines IL-17 and IL-6, and chemokine CXCL10 in retina ^10^ |
| Diabetic retinopathy | HRECs under high glucose | Pericyte-like differentiated phenotype of ASCs | Increase transendothelial electrical resistance, expression of adherens junction and tight junction proteins;  Preserve BRB integrity | Inhibit phospho-ERK1/2/phospho-cPLA2/COX-2 pathway | Reduce inflammatory cytokines TNF-α, IL-1β, and MMP-9; Reduce angiogenic factor VEGF and in fibrotic TGF-β1^11^ |
| Diabetic retinopathy | HRECs under high glucose | Human umbilical cord MSCs-derived exosomes | Reduce hyperglycemia-induced retinal inflammation | Suppressed HMGB1 signaling pathway | Decrease the levels of caspase-1, interleukin-1β (IL-1β) and IL-18; Reduce the activity of the NLRP3 inflammasome ^12^ |
| Diabetic retinopathy | HRECs under high glucose | Immortalized adipose derived MSCs-derived exosomes | Repair of high glucose-induced retinal vascular endothelial cell damage | Not mentioned | Reduce the mRNA levels of IL-1β, TNF-α^13^ |
| Diabetic retinopathy |  | MSC-derived small extracellular vesicles (sEVs) | Suppress microglial activation;  Improve histological morphology and blood-retinal barrier function | Inhibit NLRP3 inflammasome activation | Reduce inflammatory cytokine: IL-6, MCP-1, and TNF-α^14^ |
| Diabetic hepatopathy | HFD‐induced diabetic mice | MSCs-derived apoptotic vesicles | Alleviation of glucose intolerance and insulin resistance | Increase calreticulin (CRT)-mediated efferocytosis | Inhibit macrophage accumulation and transformate macrophages towards an anti-inflammation phenotype in liver ^15^ |
| Diabetic foot ulcer | STZ-induced diabetic rats | BMSCs | Promote collagen deposition, nucleated cell proliferation, neovascularization, and enhanced diabetic wound healing | Not mentioned | Inhibiting the expression of M1 macrophages; Promote the expression of M2 macrophages^16^ |
| Diabetic myopathy | HFD fed db/db mice | Human umbilical cord MSCs-derived exosomes | Increase grip strength, running endurance, and muscle mass with elevated CSA of muscle fibres;  Reduce Atrogin1 and MuRF1 expression | Activate AMPK/ULK1 signaling | Enhance autophagy ^17^ |
| Diabetic peripheral neuropathy | db/db mice | MSCs-derived exosomes | Decreased the threshold for thermal and mechanical stimuli and increased nerve conduction velocity in diabetic mice. | Toll-like receptor (TLR)4/NF-κB signaling pathway | Decreased M1 and increased M2 macrophage phenotype markers; Suppress proinflammatory cytokines^18^ |
| Diabetic peripheral neuropathy | db/db mice | Engineered MSC-exosomes loaded with miR-146a (exo-146a) | Increase nerve conduction velocity, decrease and thermal and mechanical stimuli threshold | Inhibite TLR-4/NF-κB signaling pathway | Suppress the peripheral blood inflammatory monocytes and the activation of endothelial cells ^19^ |
| Diabetic polyneuropathy | db/db mice | Conditioned medium from MSC cultures | Improve thermal and mechanical sensitivity, restoring intraepidermal nerve fiber density, reducing neuron and Schwann cell apoptosis, | Not mentioned | Improving angiogenesis, and reduce chronic inflammation of peripheral nerves^20^ |
| Diabetic neuropathy | STZ-induced diabetic mice | Conditioned medium from MSC cultures | Reverse the degenerative changes in axons and myelin sheath, and reduced area and density of unmyelinated fibers | Not mentioned | Inhibit multiple parameters of spinal neuroinflammation; reduce of activated astrocytes and microglia, oxidative stress signals, galectin-3, IL-1β, and TNF-α production; Increased the levels of anti-inflammatory cytokines, IL-10, and TGF-β ^21^ |

Abbreviations: ACR, albumin to creatinine ratio; ASCs, adipose mesenchymal stem cells; BMSCs, bone marrow mesenchymal stem cells; BMSC-EVs, Bone marrow-derived mesenchymal stem cell-derived extracellular vesicles; BUN, blood urea nitrogen; CD163: Cluster of Differentiation 163; COX-2, cyclooxygenases-2; GSDMD, Gasdermin D; HFD, a high-fat diet; hESC, human embryonic stem cell; HRECs, human retinal endothelial cells; IL, interleukin; iNOS, inducible nitric oxide synthase; LXA4-ALX/FPR2, lipoxin A4-ALX/formyl peptide receptor type 2; MCP-1, monocyte chemoattractant protein-1; MMP, matrix metalloproteinase; MSCs, mesenchymal stem cells; NF-κB, Nuclear factor kappa B; NLRP3,nucleotide-binding oligomerization domain, leucine-rich repeat and pyrin domain-containing protein 3; PD-1, programmed cell death protein 1; PD-L1, programmed cell death ligand 1; PLA2, phospholipases A2; Scr, serum creatinine; ROS, reactive oxygen species; STZ, Streptozotocin; STAT1, signal transducer and activator of transcription;TRAF6, tumor necrosis factor receptor-associated factor-6; TNF-α, tumor necrosis factor-α; TGF-β, transforming growth factor β; TLR, Toll-like receptor; VEGF, vascular endothelial growth factor.

**Table S3. Clinical trials of prescription formulas for diabetic complications.**

| TCM  (Trial registration) | Main Components | Indications | Number of  enrolled  patients | Intervention | Study  Duration  (Months) | Results |
| --- | --- | --- | --- | --- | --- | --- |
| Huangkui capsule  (NCT03016832) ^22^ | Ethanol extract of flowers in *Abelmoschus manihot* | DKD | 413 | irbesartan vs. *A. manihot* vs. combined treatment | 6 | *A. manihot*, in the form of Huangkui capsule, combined with irbesartan is an effective therapy for T2D patients with DKD in reduction of albuminuria and proteinuria. |
| Liu‒wei‒di‒huang‒wan  (NCT02488252) ^23^ | *Radix*-*Rehmannia*, *Fructus*-*Corni*, *Rhizoma*-*Dioscoreae*, *Poria*, *Cortex*-*Moutan*, and *Rhizoma*-*Alismatis* | DKD | 148 | Add-on Liu-wei-di-huang-wan vs. standard care alone | 12 | 48 weeks of add-on Rehmannia-6–based Chinese medicine treatment independently stabilized eGFR and could be a useful strategy in the multidisciplinary management of patients with T2D and CKD. |
| Zicuiyin decoction  (ChiCTR-OON-17012076) ^24^ | Astragalus, Rehmannia glutinosa, *Dioscorea oppositifolia*, *Cornus officinalis* | DKD | 88 | Zicuiyin decoction vs. Huangkui capsule | 2 | Zicuiyin had better efficacy in improving and protecting kidney function. It would be an alternative option to treat DKD, especially those who decline eGFR and gut microbiota dysbiosis. |
| Qidan Tangshen Granule (ChiCTR2200056016) ^25^ | astragalus, prepared Rehmannia root, cornelian cherry, salvia root, prepared aconite root, Poria, Chinese yam, Szechuan lovage root, angelica root, zedoary, and motherwort. | DKD | 219 | Qidan Tangshen Granule vs. conventional treatment. | 12 | Qidan Tangshen Granule treatment effectively reduces oxidative stress, improves glycemic control, ameliorates kidney function, and leads to favorable clinical outcomes in DKD patients with T2D. |
| Keluoxin (KLX) capsules (ChiCTR2100052764) ^26^ | Astragali radix, Pseudostellariae radix, Lycii fructus, Ligustri lucidi fructus, Rhei radix et rhizome, Hirudo. | DKD | 129 | KLX (6 g/day) and irbesartan tablets (150 mg/day) vs. KLX placebo (6 g/day) and irbesartan tablets (150 mg/day). | 6 | KLX capsules combined with irbesartan can reduce microalbuminuria, relieve the symptoms, and improve the quality of life for patients with T2D and DKD compared with the use of irbesartan alone. |
| Tangbi Waixi (TW) Decoction  (ChiCTR-IOR-16009331) ^27^ | Seven herbal drugs that can activate the flow of Qi and disperse blood stasis | DPN | 632 | Foot baths with TW herbal granules 66.9 g vs. 6.69 g | 3 | TW foot bath therapy was safe and significantly benefitted the patients with DPN, while a low dose of TW appeared to be more effective than a high dose. |
| Xiaoketongbi Formula (XF) (ChiCTR2200056006) ^28^ | Peach kernel, *Rheum officinale*, *Astragalus mongholicus*, *Angelica sinensis*, and *Salvia* | DPN | 68 | Xiaoketongbi Formula vs. pregabalin | 2.5 | XF is equivalent to pregabalin in reducing pain symptoms and improves the quality of life in patients with PDN. In addition, XF has the potential to improve nerve function by increasing nerve conduction velocity. |
| Tangbi Formula (ChiCTR-IOR-15006626) ^29^ | Astragalus membranaceus, cassia twig, Ligusticum chuanxiong Hort, Radix Paeoniae Alba, and Caulis Spatholobi. | DSPN | 188 | Tangbi Formula plus methylcobalamin vs. placebo plus methylcobalamin | 6 | Combining Tangbi Formula with basal therapy can be safer and more effective in improving the symptoms of DSPN patients |

Abbreviations: DKD, diabetic kidney disease; DPN, Diabetic peripheral neuropathy; DSPN, diabetic sensorimotor peripheral neuropathy; eGFR, estimated glomerular filtration rate; KLX, Keluoxin; T2D, type 2 diabetes; TCM, traditional Chinese medicine;TW, Tangbi Waixi; XF, Xiaoketongbi Formula.

**Table S4. The references of clinical trials in Table 2.**

| Drug name | Clinical trials’ number (Phase) | Reference |
| --- | --- | --- |
| Dorzagliatin | NCT03141073 (phase III) | Yang, W. et al. Dorzagliatin add-on therapy to metformin in patients with type 2 diabetes: a randomized, double-blind, placebo-controlled phase 3 trial. Nat Med. 28, 974-981 (2022). |
| Mazdutide | NCT05606913 (phase III, no results); | Efficacy and Safety of IBI362 Versus Dulaglutide as add-on to Metformin and/or SGLT2 Inhibitor or TZD in Subjects With Type 2 Diabetes (DREAMS-2). 2022. https://clinicaltrials.gov/study/NCT05606913 |
|  | NCT05607680 (phase III, no results) | A Randomized, Double-blind, Placebo-controlled Phase III Study Evaluating the Efficacy and Safety of IBI362 in Chinese Participants With Obesity or Overweight (GLORY-1). 2022. https://clinicaltrials.gov/study/NCT05607680 |
| Retatrutide | NCT06354660 (phase III, no results); | A Phase 3, Randomized, Multicenter, Double-Blind Study to Investigate the Efficacy and Safety of Retatrutide Once Weekly Compared With Placebo in Adult Participants With Type 2 Diabetes and Inadequate Glycemic Control With Diet and Exercise Alone (TRANSCEND-T2D-1). 2024. https://clinicaltrials.gov/study/NCT06354660 |
|  | NCT06662383 (phase III, no results) | A Phase 3, Randomized, Double-Blind Study to Evaluate the Efficacy and Safety of Retatrutide Compared to Tirzepatide in Adults Who Have Obesity. 2024. https://clinicaltrials.gov/study/NCT06662383 |
| CagriSema | NCT06534411 (phase III，no results) | Efficacy and Safety of Co-administered Cagrilintide and Semaglutide (CagriSema) 1.0 mg/1.0 mg s.c. Once Weekly Versus Tirzepatide 5 mg s.c. Once Weekly in Participants With Type 2 Diabetes Inadequately Controlled on Metformin, SGLT2 Inhibitor or Both. 2024. https://clinicaltrials.gov/study/NCT06534411 |
|  | NCT06780449 (phase III，no results) | Long-term Efficacy and Safety of Cagrilintide s.c. 2.4 mg in Combination With Semaglutide s.c. 2.4 mg (CagriSema 2.4 mg/2.4 mg) Once Weekly Versus Placebo in Participants With Obesity. 2025. https://clinicaltrials.gov/study/NCT06780449 |
|  | NCT05669755 (phase III，no results) | The Cardiovascular Safety and Efficacy of Cagrilintide 2.4 mg s.c. in Combination With Semaglutide 2.4 mg s.c. (CagriSema 2.4 mg/2.4 mg s.c.) Once-weekly in Participants With Established Cardiovascular Disease. 2022. https://clinicaltrials.gov/study/NCT05669755 |
|  | NCT06131372 (phase II，no results) | Efficacy and Safety of Co-administered Cagrilintide and Semaglutide (CagriSema 2.4 mg/2.4 mg) Once Weekly Versus Semaglutide 2.4 mg, Cagrilintide 2.4 mg and Placebo in People With Chronic Kidney Disease and Type 2 Diabetes Living With Overweight or Obesity. 2023. https://clinicaltrials.gov/study/NCT06131372 |
| Dapansutrile | NCT06047262 (phase II, no result) | A Multi-Centre, Randomised, Double-Blind, Placebo-Controlled Trial of the Safety and Efficacy of the Oral NLRP3 Inhibitor Dapansutrile in Subjects With Type 2 Diabetes Mellitus. 2023. https://clinicaltrials.gov/study/NCT06047262 |
| HD-6277 | NCT06647550 (phase II, no result) | A Multi-center, Double-blind, Randomized, Placebo-controlled, Parallel-group, Dose-finding, Phase 2 Study to Assess the Efficacy and Safety of HD-6277 in Adult Patients With Inadequate Control of Type 2 Diabetes Mellitus by Diet and Exercise. 2024. https://clinicaltrials.gov/study/NCT06647550 |
| SER-150 | NCT04881123 (phase II/III, no results) | Randomized, Double-blind, Placebo-controlled, Parallel Groups, Multicenter Pivotal Study Assessing the Efficacy and Safety of 15 mg Twice a Day (BID) of SER150 in Well-controlled Type 2 Diabetic Patients With Diabetic Kidney Disease and Albuminuria in Treatment With an Angiotensin Converting Enzyme Inhibitor or an Angiotensin Receptor Antagonist. 2021. https://clinicaltrials.gov/study/NCT04881123 |
| Selonsertib | NCT04026165 (phase IIb) | Heerspink, H. J. L. et al. Selonsertib in Patients with Diabetic Kidney Disease: A Phase 2b Randomized Active Run-In Clinical Trial. Journal of the American Society of Nephrology : JASN 12,1726-1736, (2024). |
| Bremelanotide | NCT05709444 (phase IIb, no results) | A Phase IIb, Multicenter, Open-Label, Prospective Study of Bremelanotide in Diabetic Kidney Disease to Assess the Efficacy in Reducing Urinary Protein and Maintaining Podocyte Density and Function. 2023. https://clinicaltrials.gov/study/NCT05709444 |
| Avenciguat | NCT04750577 (phase II) | Heerspink, H. J. L. et al. Effect of Avenciguat on Albuminuria in Patients with CKD: Two Randomized Placebo-Controlled Trials. Journal of the American Society of Nephrology : JASN 35, 1227-1239 (2024). |
| R3R-01 | NCT06600412 (phase II, no results) | Effects of the ABCA1 Inducer R3R01 on Albuminuria Levels in Diabetic Kidney Disease. 2024. https://clinicaltrials.gov/study/NCT06600412 |
| SC-0062 | NCT05687890 (phase II, no results) | A Randomized, Double Blind, Placebo Parallel Controlled, 2 Cohorts, Multicenter Phase II Study to Investigate the Safety and Efficacy of SC0062 Capsule in Patients With Chronic Kidney Disease With Albuminuria. 2023. https://clinicaltrials.gov/study/NCT05687890 |
| Inclisiran | NCT03399370 (phase III);  NCT03400800 (phase III)  NCT03397121 (phase III) | Leiter, L. A. et al. Inclisiran in individuals with diabetes or obesity: Post hoc pooled analyses of the ORION-9, ORION-10 and ORION-11 Phase 3 randomized trials. Diabetes Obes Metab 26, 3223-3237 (2024) |
| Bempedoic acid | NCT02993406 (phase III) | Ray, K. K. et al. Efficacy and safety of bempedoic acid among patients with and without diabetes: prespecified analysis of the CLEAR Outcomes randomised trial. Lancet Diabetes Endocrinol. 12, 19-28 (2024). |
| MEDI6570 | NCT05912218 (phase II, no results) | REducing Diabetic MacrovascUlar Complications DuE to Peripheral Arterial Disease- REDUCE-PAD. 2023. https://clinicaltrials.gov/study/NCT05912218 |
| Pentoxifylline | EudraCT #2009-016595-77 (not applicable) | Donate-Correa, J. et al. Pentoxifylline ameliorates subclinical atherosclerosis progression in patients with type 2 diabetes and chronic kidney disease: a randomized pilot trial. Cardiovasc Diabetol. 23, 314 (2024). |
| Alpha-lipoic acid | NCT06056687 (not applicable, no result) | Effect of Alpha-lipoic Acid on Inflammatory and Cardiac Fibrosis Markers in Diabetic Patients With Ischemic Cardiomyopathy. 2023. https://clinicaltrials.gov/study/NCT06056687 |
| OCS-01 | NCT06172257 (phase III, no results) | A Phase 3 Double-masked, Randomized, Multicenter Study of the Efficacy and Safety of OCS 01 Eye Drops in Subjects with Diabetic Macular Edema. 2023. https://clinicaltrials.gov/study/NCT06172257 |
| Aflibercept | NCT04429503 (phase II/III) | Brown, D. M. et al. Intravitreal aflibercept 8 mg in diabetic macular oedema (PHOTON): 48-week results from a randomised, double-masked, non-inferiority, phase 2/3 trial. Lancet 403, 1153-1163 (2024). |
| Restoret | NCT06571045 (phase II/III, no results) | A Randomized, Double-Masked, Multi-Center, 3-Arm Pivotal Phase 2/3 Study to Evaluate The Efficacy and Safety of Intravitreal EYE103 Compared With Intravitreal Ranibizumab (0.5mg) in Participants With Diabetic Macular Edema. 2024. https://clinicaltrials.gov/study/NCT06571045 |
| RC-28 | NCT05885503 (phase III, no results) | A Phase III, Multicenter, Randomized, Double-blind, Active Controlled Trial of RC28-E Intravitreal Injection in Subjects With Diabetic Macular Edema. 2023. https://clinicaltrials.gov/study/NCT05885503 |
| Vamikibart | NCT05151731 (phase II, no result) | A Phase II, Multicenter, Randomized, Double Masked, Active Comparator-Controlled Study to Investigate the Efficacy, Safety, Tolerability, Pharmacokinetics, and Pharmacodynamics of RO7200220 Administered Intravitreally in Patients With Diabetic Macular Edema. 2021. https://clinicaltrials.gov/study/NCT05151731 |
| BI-764524 | NCT06321302 (phase II, no result) | CRIMSON: A Multicentre, Randomised, Sham-controlled (and Active Controlled in the USA), Double-masked, 72-week Trial to Study the Safety, Tolerability, Pharmacokinetics, and Efficacy of 3 Dosing Regimens of Intravitreal BI 764524 in Patients With Moderately Severe to Severe Non-proliferative Diabetic Retinopathy. 2024. https://clinicaltrials.gov/study/NCT06321302 |
| Lanifibranor | NCT03459079 (phase II) | Barb, D. et al. Pan-PPAR agonist lanifibranor improves insulin resistance and hepatic steatosis in patients with T2D and MASLD. J Hepatol 82, 979-991 (2025). . |
| PF-06835919 | NCT03969719 (phase II) | A PHASE 2A, RANDOMIZED, DOUBLE-BLIND, PLACEBO-CONTROLLED, 3-ARM, PARALLEL GROUP STUDY TO EVALUATE SAFETY, TOLERABILITY AND PHARMACODYNAMICS OF PF-06835919 ADMINISTERED DAILY FOR 16 WEEKS IN ADULTS WITH NON-ALCOHOLIC FATTY LIVER DISEASE AND TYPE 2 DIABETES MELLITUS ON METFORMIN. 2019. https://clinicaltrials.gov/study/NCT03969719 |
| Tipelukast | NCT05464784 (phase II, no results) | A Phase 2, Double-Blind, Randomized, Placebo-Controlled Study to Evaluate the Safety, Tolerability and Efficacy of MN-001 in Patients Diagnosed With Non-alcoholic Fatty Liver Disease, Type 2 Diabetes Mellitus, and Hypertriglyceridemia. 2022. https://clinicaltrials.gov/study/NCT05464784 |
| MET-409 | NCT04702490 (phase II, no results) | A Phase 2A Study to Evaluate MET409 Alone or in Combination With Empagliflozin in Patients With Type 2 Diabetes Mellitus (T2DM) and Nonalcoholic Steatohepatitis (NASH). 2021. https://clinicaltrials.gov/study/NCT04702490 |
| Tirzepatide | NCT05751720 (phase I/II, no results) | Effect on Non-Alcoholic Fatty Liver Disease With Advanced Fibrosis in Patients With Type 2 Diabetes Mellitus on Treatment With Gastric Inhibitory Polypeptide / Glucagon Like Peptide-1 Analogue (Tirzpatide). 2023. https://clinicaltrials.gov/study/NCT05751720 |
| Acetyllevocarnitine Hydrochloride | NCT05319275 (phase III) | Guo, L. et al. Acetyllevocarnitine Hydrochloride for the Treatment of Diabetic Peripheral Neuropathy: A Phase 3 Randomized Clinical Trial in China. Diabetes 73, 797-805 (2024). |
| HSK16149 | NCT04647773 (phase III) | Guo, X. et al. GABA Analogue HSK16149 in Chinese Patients With Diabetic Peripheral Neuropathic Pain: A Phase 3 Randomized Clinical Trial. JAMA Netw Open 7, e2425614 (2024). |
| Oxybutynin | NCT03050827 (phase III) | Casselini, C. M. et al. A muscarinic receptor antagonist reverses multiple indices of diabetic peripheral neuropathy: preclinical and clinical studies using oxybutynin. Acta Neuropathol 147, 60 (2024). |
| Suzetrigine | NCT06696443 (phase III, no results) | A Phase 3, Open-label Study Evaluating the Long-term Safety and Effectiveness of Suzetrigine in Subjects With Pain Associated With Diabetic Peripheral Neuropathy. 2024. https://clinicaltrials.gov/study/NCT06696443 |
| ISC-17536 | NCT01726413 (phase II) | Jain, S. M. et al. Randomized, double-blind, placebo-controlled trial of ISC 17536, an oral inhibitor of transient receptor potential ankyrin 1, in patients with painful diabetic peripheral neuropathy: impact of preserved small nerve fiber function. Pain 163, e738-e747 (2022). |
| LX-9211 | NCT04455633 (phase II) | Pop-Busui, R. et al. Efficacy and Safety of LX9211 for Relief of Diabetic Peripheral Neuropathic Pain (RELIEF-DPN 1): Results of a Double-Blind, Randomized, Placebo-Controlled, Proof-of-Concept Study. Diabetes Care 47, 1325-1332 (2024). |
| Topical Esmolol Hydrochloride | NCT03998436 (phase III) | Rastogi, A. et al. Topical Esmolol Hydrochloride as a Novel Treatment Modality for Diabetic Foot Ulcers: A Phase 3 Randomized Clinical Trial. JAMA Netw Open 6, e2311509 (2023). |
| ENERGI-F703 | NCT05930210 (phase III, no results) | A Randomized, Double-Blind, Vehicle-controlled, Parallel, Phase III Study to Evaluate Efficacy and Safety of ENERGI-F703 GEL in Subjects with Diabetic Foot Ulcers. 2023. https://clinicaltrials.gov/study/NCT05930210 |
| TP-102 | NCT05948592 (phase II, no result) | A Phase 2 CT to Evaluate the Safety and Efficacy of Topical Administration of Bacteriophage Therapy TP-102 in Patients With DFI. 2023. https://clinicaltrials.gov/study/NCT05948592 |
| Pravibismane | NCT05174806 (phase II, no result) | A Randomized, Open Label, Controlled, Phase 2, Multi-Center Study to Assess Safety, Tolerability and Efficacy of Adjunctive Treatment With Topically Applied MBN-101 (Pravibismane Topical Suspension) in Subjects With Moderate Diabetic Foot Infection (DFI). 2021. https://clinicaltrials.gov/study/NCT05174806 |
| ILP-100 | NCT05608187 (phase II, no result) | A Randomized, Double-Blind, Placebo-controlled, Parallel, Exploratory Phase 2a Study to Evaluate Safety and Biological Effect on Wound Healing of ILP100-Topical in Subjects With Diabetic Foot Ulcers. 2022. https://clinicaltrials.gov/study/NCT05608187 |

References

1 Wang, J. *et al.* Human placenta-derived mesenchymal stem cells ameliorate diabetic kidney disease by modulating the T helper 17 cell/ regulatory T-cell balance through the programmed death 1 / programmed death-ligand 1 pathway. *Diabetes Obes Metab* **26**, 32-45 (2024).

2 Zhang, Y. *et al.* MicroRNA-146a-5p-modified human umbilical cord mesenchymal stem cells enhance protection against diabetic nephropathy in rats through facilitating M2 macrophage polarization. *Stem Cell Res Ther* **13**, 171 (2022).

3 Yuan, Y. *et al.* Mesenchymal stem cells elicit macrophages into M2 phenotype via improving transcription factor EB-mediated autophagy to alleviate diabetic nephropathy. *Stem Cells* **38**, 639-652 (2020).

4 Xiang, E. *et al.* Human umbilical cord-derived mesenchymal stem cells prevent the progression of early diabetic nephropathy through inhibiting inflammation and fibrosis. *Stem Cell Res Ther* **11**, 336 (2020).

5 Wang, Y. *et al.* Human umbilical cord mesenchymal stem cells attenuate podocyte injury under high glucose via TLR2 and TLR4 signaling. *Diabetes Res Clin Pract* **173**, 108702 (2021).

6 Yu, S. *et al.* Treatment with adipose tissue-derived mesenchymal stem cells exerts anti-diabetic effects, improves long-term complications, and attenuates inflammation in type 2 diabetic rats. *Stem Cell Res Ther* **10**, 333 (2019).

7 Abel, E. D. *et al.* Diabetes mellitus-Progress and opportunities in the evolving epidemic. *Cell* **187**, 3789-3820 (2024).

8 Yang, Q., Chen, Q., Li, S. & Luo, J. Mesenchymal stem cells ameliorate inflammation and pyroptosis in diabetic cardiomyopathy via the miRNA-223-3p/NLRP3 pathway. *Diabetol Metab Syndr* **16**, 146 (2024).

9 Liu, L. *et al.* Bone marrow mesenchymal stem cell-derived extracellular vesicles alleviate diabetes-exacerbated atherosclerosis via AMPK/mTOR pathway-mediated autophagy-related macrophage polarization. *Cardiovasc Diabetol* **24**, 48 (2025).

10 Rong, L. *et al.* Clinical-grade human embryonic stem cell-derived mesenchymal stromal cells ameliorate diabetic retinopathy in db/db mice. *Cytotherapy* **26**, 606-615 (2024).

11 Lupo, G. *et al.* Protective Effects of Human Pericyte-like Adipose-Derived Mesenchymal Stem Cells on Human Retinal Endothelial Cells in an In Vitro Model of Diabetic Retinopathy: Evidence for Autologous Cell Therapy. *Int J Mol Sci* **24**, 913, (2023).

12 Zhang, W., Wang, Y. & Kong, Y. Exosomes Derived From Mesenchymal Stem Cells Modulate miR-126 to Ameliorate Hyperglycemia-Induced Retinal Inflammation Via Targeting HMGB1. *Invest Ophthalmol Vis Sci* **60**, 294-303 (2019).

13 Wu, S. *et al.* Research on the role of exosomes secreted by immortalized adipose-derived mesenchymal stem cells differentiated into pericytes in the repair of high glucose-induced retinal vascular endothelial cell damage. *Exp Eye Res* **247**, 110046 (2024).

14 Chen, Y. *et al.* MSC-Derived Small Extracellular Vesicles Alleviate Diabetic Retinopathy by Delivering miR-22-3p to Inhibit NLRP3 Inflammasome Activation. *Stem Cells* **42**, 64-75 (2024).

15 Zheng, C. *et al.* Apoptotic vesicles restore liver macrophage homeostasis to counteract type 2 diabetes. *J Extracell Vesicles* **10**, e12109 (2021).

16 Bai, H. *et al.* Regulation of inflammatory microenvironment using a self-healing hydrogel loaded with BM-MSCs for advanced wound healing in rat diabetic foot ulcers. *J Tissue Eng* **11**, 2041731420947242 (2020).

17 Song, J. *et al.* Mesenchymal stromal cells ameliorate diabetes-induced muscle atrophy through exosomes by enhancing AMPK/ULK1-mediated autophagy. *J Cachexia Sarcopenia Muscle* **14**, 915-929 (2023).

18 Fan, B. *et al.* Mesenchymal stromal cell-derived exosomes ameliorate peripheral neuropathy in a mouse model of diabetes. *Diabetologia* **63**, 431-443 (2020).

19 Fan, B., Chopp, M., Zhang, Z. G. & Liu, X. S. Treatment of diabetic peripheral neuropathy with engineered mesenchymal stromal cell-derived exosomes enriched with microRNA-146a provide amplified therapeutic efficacy. *Exp Neurol* **341**, 113694 (2021).

20 De Gregorio, C. *et al.* Human adipose-derived mesenchymal stem cell-conditioned medium ameliorates polyneuropathy and foot ulceration in diabetic BKS db/db mice. *Stem Cell Res Ther* **11**, 168 (2020).

21 Evangelista, A. F. *et al.* Bone marrow-derived mesenchymal stem/stromal cells reverse the sensorial diabetic neuropathy via modulation of spinal neuroinflammatory cascades. *J Neuroinflammation* **15**, 189 (2018).

22 Zhao, J. *et al.* Efficacy of Combined Abelmoschus manihot and Irbesartan for Reduction of Albuminuria in Patients With Type 2 Diabetes and Diabetic Kidney Disease: A Multicenter Randomized Double-Blind Parallel Controlled Clinical Trial. *Diabetes Care* **45**, e113-e115 (2022).

23 Chan, K. W. *et al.* Add-on Rehmannia-6-Based Chinese Medicine in Type 2 Diabetes and CKD: A Multicenter Randomized Controlled Trial. *Clin J Am Soc Nephrol* **18**, 1163-1174 (2023).

24 Liu, J. *et al.* Efficacy and safety of Zicuiyin decoction on diabetic kidney disease: A multicenter, randomized controlled trial. *Phytomedicine* **100**, 154079 (2022).

25 Yang, H. *et al.* Effects of Qidan Tangshen Granule on diabetic kidney disease in patients with type 2 diabetes. *Diabetes Res Clin Pract* **209**, 111128 (2024).

26 Zhao, J. *et al.* A Prospective, Multicentered, Randomized, Double-Blind, Placebo-Controlled Clinical Trial of Keluoxin Capsules in the Treatment of Microalbuminuria in Patients with Type 2 Early Diabetic Kidney Disease. *J Integr Complement Med* **30**, 185-195 (2024).

27 Huang, H. Y. *et al.* Effect and Safety of Herbal Medicine Foot Baths in Patients with Diabetic Peripheral Neuropathy: A Multicenter Double-Blind Randomized Controlled Trial. *Chin J Integr Med* **30**, 195-202 (2024).

28 Lu, Q. *et al.* Xiaoketongbi Formula vs pregabalin for painful diabetic neuropathy: A single-center, randomized, single-blind, double-dummy, and parallel controlled clinical trial. *J Diabetes* **14**, 551-561 (2022).

29 Zhao, X. *et al.* TangBi Formula for Painful Diabetic Distal Symmetric Polyneuropathy: A Multicenter, Randomized, Double-Blind, Placebo-Controlled and Parallel-Group Trial. *J Diabetes* **17**, e70045 (2025).
